# Molecular Display of the Animal Meta-Venome for Discovery of Novel Therapeutic Peptides

**DOI:** 10.1016/j.mcpro.2024.100901

**Published:** 2024-12-31

**Authors:** Meng-Hsuan Hsiao, Yang Miao, Zixing Liu, Konstantin Schütze, Nathachit Limjunyawong, Daphne Chun-Che Chien, Wayne Denis Monteiro, Lee-Shin Chu, William Morgenlander, Sahana Jayaraman, Sung-eun Jang, Jeffrey J. Gray, Heng Zhu, Xinzhong Dong, Martin Steinegger, H. Benjamin Larman

**Affiliations:** 1 Institute for Cell Engineering, Division of Immunology, Department of Pathology, Johns Hopkins University School of Medicine, Baltimore, Maryland, USA; 2 Department of Chemical and Biomolecular Engineering, Whiting School of Engineering, Johns Hopkins University, Baltimore, Maryland, USA; 3 Department of Biomedical Engineering, Whiting School of Engineering, Johns Hopkins University, Baltimore, Maryland, USA; 4 Department of Biology, Zanvyl Krieger School of Arts and Sciences, Johns Hopkins University, Baltimore, Maryland, USA; 5 School of Biological Sciences, Seoul National University, Seoul, South Korea; 6 The Solomon H. Snyder Department of Neuroscience, Johns Hopkins University School of Medicine, Baltimore, Maryland, USA; 7 Center of Research Excellence in Allergy and Immunology, Research Department, Faculty of Medicine Siriraj Hospital, Mahidol University, Bangkok, Thailand; 8 Department of Pharmacology and Molecular Sciences, Johns Hopkins University School of Medicine, Baltimore, Maryland, USA; 9 Viral Oncology Program, Department of Oncology, The Sidney Kimmel Comprehensive Cancer Center, Johns Hopkins University School of Medicine, Baltimore, Maryland, USA; 10 Howard Hughes Medical Institute, Johns Hopkins University School of Medicine, Baltimore, Maryland, USA; 11 Artificial Intelligence Institute, Seoul National University, Seoul, South Korea; 12 Institute of Molecular Biology and Genetics, Seoul National University, Seoul, South Korea

**Keywords:** venom, phage display, ligand discovery, database mining, itch receptor

## Abstract

Animal venoms, distinguished by their unique structural features and potent bioactivities, represent a vast and relatively untapped reservoir of therapeutic molecules. However, limitations associated with comprehensively constructing and expressing highly complex venom and venom-like molecule libraries have precluded their therapeutic evaluation *via* high-throughput screening. Here, we developed an innovative computational approach to design a highly diverse library of animal venoms and “metavenoms”. We used programmable M13 hyperphage display to preserve critical disulfide-bonded structures for highly parallelized single-round biopanning with quantitation *via* high-throughput DNA sequencing. Our approach led to the discovery of Kunitz-type domain containing proteins that target the human itch receptor Mas-related G-protein coupled receptor member X4, which plays a crucial role in itch perception. Deep learning-based structural homology mining identified two endogenous human homologs, tissue factor pathway inhibitor (TFPI), and serine peptidase inhibitor, Kunitz type 2 (SPINT2), which exhibit agonist-dependent potentiation of Mas-related G-protein coupled receptor member X4. Highly multiplexed screening of animal venoms and metavenoms is therefore a promising approach to uncover new drug candidates.

## Highlights

• Designed diverse venom peptide and "metavenom" peptide scaffold libraries.
• Developed sequencing-assisted single-round M13 hyperphage display panning workflow.
• Presented an approach to nominate venom peptide scaffolds for lead optimization.
• Discovered novel endogenous potentiators of the human itch receptor MRGPRX4.

## Graphical Abstract

**Figure:**
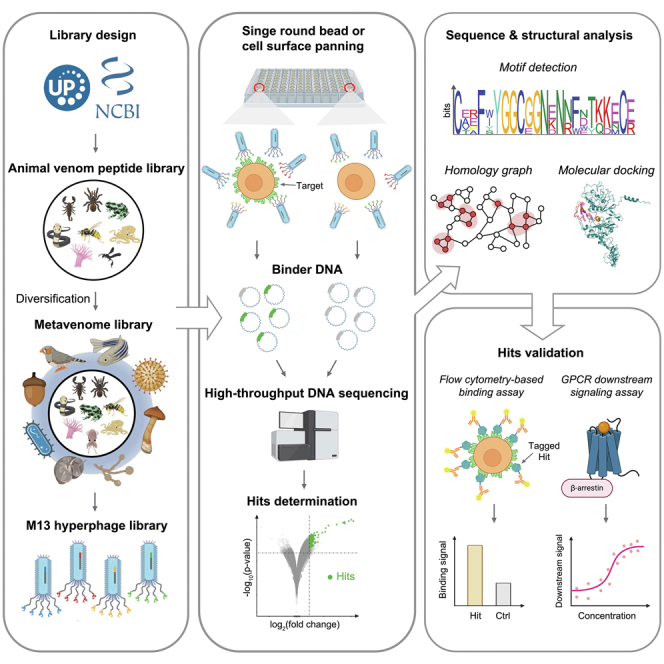

## In Brief

Animal venom peptides, often with potent and selective bioactivities, serve as a rich and diverse source of molecular scaffolds. Here, we design and construct a diverse meta-venome library using M13 hyperphage display to preserve critical disulfide-bond structures. A DNA sequencing-assisted, single-round biopanning workflow is presented, which is used to identify known and novel ligands for the epidermal growth factor receptor and the human itch receptor. This platform provides an efficient path to discovering new venom peptide-derived therapeutic lead molecules.

Animal venoms have emerged as a vast reservoir of bioactive molecules with relatively untapped potential for drug discovery. These naturally occurring toxins, with their high potency, selectivity, and unique structural features, have evolved over millions of years to target a vast array of biological systems (1, 2, 3, 4). A key feature of many animal venom (AV) polypeptides is the presence of multiple disulfide bridges, which stably lock the polypeptide chain into a single conformation, resulting in resistance to protease digestion and high binding selectivity and affinity (4, 5). Additionally, the diverse characteristics of venom polypeptides, including their small size, unique structural motifs, target specificity, and ability to modulate various biological pathways, also contribute significantly to their value in biological research and drug development. Recent, well-known successes of toxin-derived therapeutics include ziconotide (4, 6), an analgesic derived from cone snail venom, as well as exenatide (4, 6) and tirzepatide (7, 8), antidiabetic drugs derived from Gila monster venom. In addition, venom-derived drugs have been successfully developed for a variety of clinical applications, including anticoagulation, tumor painting (9) and chemotherapy, underscoring the broad versatility of these natural compounds (6, 10, 11).

Membrane proteins play vital roles in numerous cellular processes and are targeted by approximately 30 to 40% of Food and Drug Administration approved drugs (12). One strategy for identifying putative therapeutics that target membrane proteins involves utilizing miniproteins, small proteins typically consisting of 20 to 100 amino acids, as scaffolds for drug development (13, 14, 15). Compounds derived from these scaffolds, such as designed ankyrin repeat proteins (DARPins), affibodies and cystine dense proteins (15, 16), can be optimized for specific therapeutic indications. Due to their compact size, stability, potency, and ease of engineering, peptides and miniproteins can provide especially attractive scaffolds for deriving novel membrane protein targeting therapeutics.

Previous high-throughput approaches to discover novel drug candidates from venoms have primarily relied on activity-guided fractionation or “sequence-based” methods (5, 17). While several collections have been constructed by leveraging existing venom peptide databases, they were limited in library size (18, 19). To construct a library of comprehensive diverse venom molecules, we extracted all 21,311 full-length, active (mature) venom peptides and proteins contained in the UniProt database (20, 21), around 50% of which are below 90 amino acids long (∼10 kDa) and can thus be encoded *via* oligonucleotide library synthesis. To further expand the structural diversity of this venom scaffold library, we identified an additional 41,136 unique venom-related molecules *via* sequence homology searching of the Big Fantastic Database (BFD) (22) and Serratus databases (23), which together contain over 2.5 billion metagenomic DNA sequences. A large fraction of the AV polypeptide universe can therefore be encoded using molecular display for high-throughput drug discovery campaigns. In this proof-of-concept study, we used the M13 hyperphage display system, as it facilitates efficient polyvalent display of proteins with disulfide bonds (24, 25, 26). Sequencing-assisted single-round screening can then be used for rapid identification of target binding interactions.

As an initial demonstration of this platform, we focused on two critical receptors with distinct structural conformations: human epidermal growth factor receptor (hEGFR) and Mas-related G-protein coupled receptor member X4 (MRGPRX4). Epidermal growth factor receptor (EGFR) has a set of well-studied ligands and can be expressed both on the cell surface and in the form of a chimeric immunoglobulin fusion protein. The studies using EGFR revealed our platform’s capability to identify binders with varying affinities and diverse binding modes within complex libraries. We further expanded the application of our platform to the human itch receptor MRGPRX4, a seven-pass transmembrane G protein–coupled receptor (GPCR) that is expressed on the plasma membrane of primary sensory neurons in the dorsal root ganglion and plays a critical role in itch sensation (27, 28). Our platform identified novel candidate ligands originating from the expanded metagenomic library, as well as human homologs, including the human venom-like cystine dense proteins, tissue factor pathway inhibitor (TFPI), and serine peptidase inhibitor, Kunitz type 2 (SPINT2), which potentiate agonism of MRGPRX4. These findings underscore the value of our highly parallel single-round biopanning platform and unique computational approach for designing and assembling highly diverse venom and venom-like libraries for drug discovery.

## Experimental Procedures

### M13 Phagemid and Linker Design

The M13 phagemid vector was derived from the pSEX81 phagemid (PROGEN, Cat No. PR3005) modified to be compatible with next-generation sequencing (NGS) platforms (Supplemental Fig. S1*A*). The protein of interest (POI) was cloned at the N-terminal side of the P3 protein with EcoRI and HindIII restriction sites, and a FLAG epitope was encoded downstream of the POI. To investigate the impact of linker length on ligand enrichment, a series of linkers with various lengths were incorporated between the POI and the FLAG tag. For the M13 and M13-30 constructs, a G4S linker was used directly after the POI. For the M13-50, M13-70, M13-181, and M13-291 constructs, combinations of PAS (29, 30, 31) and G4S linkers were used to create various linker lengths between the POI and the FLAG tag. The specific linker compositions used in each construct can be found in Supplemental Table S1.

### Animal Venom, Metavenome, and Human Secretome Library Design and Synthesis

We extracted the protein sequences for the human secretome and AV library from the UniProt database using specific search criteria. Mature and active sequences that were equal to or less than 90 amino acids were selected and reverse translated for DNA synthesis.

For the metavenome (MV) library, known AV protein sequences from UniProt dataset were used as queries for searches against two metagenomic databases: BFD and a subset of Sequence Read Archive experiments identified by Serratus. We performed MMseqs2 iterative search to identify homologous sequences. The resulting sequences were filtered through several steps to reduce the sequence set size while preserving diversity of our library.

For all three libraries, sequences less than 270 base pairs were filled with PAS linker such that the final DNA length was brought up to 300 bases when appending corresponding primer binding sequences to the 5′ and 3′ end. The sequences were all synthesized by Twist Bioscience and subsequently cloned into the M13-70 phagemid vector with EcoRI and HindIII restriction sites.

The details of library design and synthesis are described in the supplemental information.

### Library Cloning

M13-70 phagemid, serving as the vector for library cloning, was digested overnight with EcoRI and HindIII restriction enzymes (NEB Cat No. R3101, R3104). In order to dephosphorylate the ends, the digested vector was treated with phosphatase for a brief 10-min duration followed by gel purification with a 2% low melting temperature agarose (Thermo Fisher Scientific Cat No. 16500100) in 1× TAE buffer.

The oligo pool, comprising all three libraries used in this study, was reconstituted in molecular biology-grade water to a concentration of 100 ng/μl. A total of 10 ng of the reconstituted oligos was used for PCR amplification (Herculase II Fusion DNA Polymerase, Agilent Cat No. 600679). Two PCR reactions were carried out. In the first round of PCR, the desired library was selectively amplified by performing 10 cycles using primers with specific binding sequences unique to each library. The amplified PCR product was then column purified (QIAGEN Cat No. 28104). Following this, 10 second-round PCR reactions were set up, with each reaction containing 30 ng of purified first-round PCR product. A total of five cycles were conducted in the second-round PCR to introduce adaptors required for the subsequent restriction digest. Finally, the PCR-amplified product was purified again and digested with EcoRI and HindIII restriction enzymes followed by gel purification.

An adequate number of ligation reactions were prepared, each containing 50 ng of DNA (comprising both vector and insert at a 1:3 M ratio) and high-concentration T4 DNA ligase (NEB Cat No. M0202T). The ligation mix was incubated at 16 °C overnight and column-purified with molecular biology-grade water. To identify the inserts and evaluate the quality of cloning, 20 colonies from each library were picked individually and miniprepped for Sanger sequencing. Subsequent analyses showed that, on average, 50 to 60% of the clones were correct at the DNA sequence level, while 60 to 70% were accurate at the amino acid level.

### M13 Phagemid *Escherichia coli* Stock Preparation

To ensure each library member was represented by a minimum of 100 colonies, the number of reactions for phagemid DNA library transformation into TOP10F′ electrocompetent cells (Thermo Fisher Scientific, Cat No. 44–0002) was calculated accordingly. The transformed cells were incubated in S.O.C. media at 37 °C for 1 h with shaking at 250 rpm, followed by plating on LB agar plates supplemented with carbenicillin (50 mg/ml), tetracycline (5 mg/ml), and 100 mM glucose. The plates were then incubated overnight at 30 °C, and the phagemid library stock was then collected and stored in −80 °C with LB supplemented with 25% glycerol, carbenicillin (50 mg/ml), and 100 mM glucose. To generate a single clone phagemid construct, the same transformation and plating protocols were followed as for the library.

### M13 Phage Expression, Purification, and Quantification

For phage library production, the *Escherichia coli* phagemid library stock, which covered at least 100-fold of its library complexity, was diluted in prewarmed LB media supplemented with 100 μg/ml carbenicillin and 100 mM glucose to an *A*_600_ of 0.1. The culture was then incubated at 30 °C until it reached an *A*_600_ of 0.4. Upon reaching the desired absorbance, the cells were infected with hyperphage (PROGEN Cat No. PRHYPE) at a multiplicity of infection of 20 and incubated at room temperature for 15 to 20 min without shaking. The cells were subsequently incubated at 30 °C with shaking at 250 rpm for 45 min. The hyperphage-infected cell pellet was collected by centrifugation at 4000g and resuspended in 2XYT media supplemented with 100 μg/ml carbenicillin, 50 μg/ml kanamycin, and 200 μM IPTG to induce protein production. To facilitate phage library production, the cell culture was incubated for 16 h at 30 °C with shaking at 230 rpm. The bacteria were then pelleted by centrifugation at 8000 rpm and 4 °C, and the phage library was collected by filtering through a protein low binding 0.22 μm filter. To remove any residual bacterial debris, the library underwent two rounds of centrifugation at 10,000*g* for 5 min at 4 °C.

Subsequently, the phage library was concentrated to a volume at least 10 times smaller than its original volume and buffer exchanged with PBS using a 15 ml Amicon 50 kDa spin filter. A protease inhibitor (Roche Cat No. 11836170001) was added to the PBS-based concentrated phage library. To determine the number of phage particles in the solution, 100 μl of the phage library was subjected to small-scale dialysis with DNAse I buffer (10 mM Tris–HCl, 2.5 mM MgCl_2_, and 0.5 mM CaCl_2_) overnight at 4 °C. The following day, DNAse I was added to the dialyzed solution and incubated at 37 °C for 10 min to destroy any single and double-stranded DNA that was not packaged within the M13 phagemid particle.

Finally, DNAse I was heat inactivated at 75 °C, and the solution was diluted 100-fold with molecular biology-grade water before quantifying the number of phage particles using quantitative PCR.

### Metagenomic Library Annotation

In this study, we aimed to annotate protein information and assign taxonomic classifications for the metagenomic library using NCBI-BLAST+ software (version 2.13.0; ebi.ac.uk/jdispatcher/sss/ncbiblast). Peptide sequences went through a blastp search against the comprehensive NCBI nonredundant protein database (nr, as of July 2022) to find similarities in sequence. Depending on the query length, we tailored the alignment parameters accordingly. For sequences that were equal or less than 30 amino acids in length, we used the parameters: "-evalue 200,000 -max_target_seqs 10 -max_hsps 1". The e-value was adjusted to accommodate short sequences. For amino acid sequences ranging between 31 and 90, the parameters were as follows: "-evalue 1e-3 -word_size 6 -max_target_seqs 10 -max_hsps 1" (default parameters: "-evalue 10 -word_size 3 -max_target_seqs 500 -max_hsps >=1"). All other blastp parameters were maintained at default settings. We had explored more sensitive parameters, such as smaller word sizes, but ultimately selected the current parameters to achieve a fine balance between sensitivity and search runtime. The blastp output included alignment metrics, protein names and accession IDs, and taxonomy identifiers (TaxIds). Using this output, we identified the best hits based on the lowest E-value and queried the corresponding TaxIds within Taxonkit (32) to annotate the complete taxonomic lineages.

### Cell Lines and Culture

The NCI-H358 and HEK 293T-CXCR2 cell lines were generously provided from Prof. Bert Vogelstein (Department of Oncology, Pathology, and Molecular Biology and Genetics, Johns Hopkins University) and Prof. Jamie Spangler (Department of Chemical and Biomolecular Engineering, Johns Hopkins University), respectively. NCI-H358 cells were cultured in McCoy's 5A medium supplemented with 10% heat-inactivated fetal bovine serum (FBS) and 50 U/ml penicillin-streptomycin, while MDA-MB-468, MDA-MB-231 overexpressing EGFR, and HEK 293T cells overexpressing EGFR and C-X-C motif chemokine receptor 2 (CXCR2) were maintained in complete Dulbecco's modified Eagle's medium (DMEM) culture media (high-glucose DMEM supplemented with sodium pyruvate, 10% heat-inactivated FBS, and 50 U/ml penicillin-streptomycin). HTLA cells, stably expressing a tTA-dependent luciferase reporter and a β-arrestin-TEV protease fusion gene, were maintained in complete DMEM media supplemented with 2 μg/ml puromycin and 100 μg/ml hygromycin. HTLA cells stably expressing MRGPRX4-tango were maintained in complete DMEM media supplemented with 2 μg/ml puromycin, 100 μg/ml hygromycin, and 100 μg/ml Zeocin. All cell lines were incubated at 37 °C, 95% humidity, and 5% CO2.

### Generation of an EGFR-Overexpressing Stable HEK 293T Cell Line

To generate HEK 293T cells overexpressing EGFR, a lentiviral transduction method was used. The full-length EGFR gene was inserted into the pCDH lentiviral expression vector, and the lentiviral particles were produced using the pPACKH1 HIV Lentivector Packaging System (System Biosciences, Cat No. LA500A-1). In brief, 3E+06 HEK 293T cells were plated on a 10 cm dish and incubated overnight in Iscove’s modified Dulbecco’s media containing 10% FBS and 2 mM L-glutamine. On the subsequent day, 2 μg of the pCDH plasmids encoding EGFR were cotransfected with the pPACK packaging plasmid mixture into HEK 293T cells, using GeneJuice (Sigma-Aldrich, Cat No. 70967) as the transfection agent. After 48 h, the media containing EGFR lentivirus was collected, filtered through a 0.45 μm filter, and used for transduction of 1E+05 HEK 293T cells in a 24-well plate along with 8 μg/ml polybrene (Sigma-Aldrich Cat No. TR-1003-G) in 500 μl of complete DMEM. Following transduction, the cells were subjected to centrifugation at 800*g* for 30 min at 32 °C and incubated overnight at 37 °C under 5% CO_2_ conditions in a humidified incubator. The next day, the culture medium was replaced with fresh complete DMEM, and the transduced cells were collected 10 days post transduction to evaluate EGFR expression using flow cytometry. Cells exhibiting the top 10% of EGFR expression were sorted, collected, and subsequently cultured in complete DMEM.

### Flow Cytometry Analysis of Receptor Expression Level

Flow cytometry was used to evaluate the expression levels of target receptors in harvested cells. Approximately 5E+05 cells were resuspended in 1 ml fluorescence-activated cell sorting (FACS) washing buffer (1% bovine serum albumin or 2% serum in PBS without Ca^2+^ or Mg^2+^, containing 0.05% NaN_3_) and transferred to a FACS tube. Following centrifugation at 300*g* for 5 min, the supernatant was discarded. Cells were then incubated with 1 μg of primary antibody diluted in 100 μl of FACS buffer. For EGFR and CXCR2 overexpressing cells, mouse anti-EGFR mAb (Thermo Fisher Scientific, Cat No. MA5-13070) and mouse anti-CXCR2 mAb (R&D Systems, Cat No. MAB331) were used for primary antibody staining, respectively. Samples were then stained for at least 30 min on ice before washing with 2 to 5 ml FACS buffer. After two subsequent washes by centrifugation at 300*g* for 5 min at room temperature, cells were incubated with goat anti-mouse Alexa Fluor 488 mAb secondary reagent (Thermo Fisher Scientific, Cat No. A11001) for at least 30 min on ice, shielded from light. Following a final double wash with FACS buffer, cells were adjusted to a concentration of approximately 1E+06 cells/ml, and the expression levels of target receptors were analyzed using flow cytometry.

### Single-Round Cell-Based and Bead-Based Screening

Two distinct screening methods were used to identify novel ligands: a cell-based system and a receptor-Fc chimera immunoprecipitation technique. Both approaches involved incubating phage libraries with target receptors, collecting binders, performing PCR, and determining candidate hits based on NGS results. To ensure statistical validation and reproducibility, each condition was represented by triplicate samples.

In the cell-based screening method, cells overexpressing target receptors were detached with cell dissociation buffer (Corning, Cat No. 25–056-CI) and collected after passing through 40 μm cell strainer. The collected cells were washed three times with ice-cold PBS containing 1% (w/v) BSA (PBSA) and then incubated separately with the phage library in 1.5 ml Eppendorf tubes containing 1% PBSA for 30 min at 4 °C to minimize nonspecific binding. Approximately 1E+06 cells were subsequently incubated with the phage library in 1% PBSA for 4 h at 4 °C on an end-to-end rotator. After washing the cells three times with ice-cold 1% PBSA, ssDNA of the bound phages was collected using a modified protocol from the Quick-DNA/RNA Miniprep Kit (Zymo Cat No. D7011). Briefly, after cell lysis and chromosomal DNA removal, RNAse A (Thermo Fisher Scientific, Cat No. EN0531) was added to digest cellular RNA at 37 °C for 30 min. Next, the RNAse-digested solution was mixed thoroughly with an equal volume of 40% molecular biology-grade ethanol for ssDNA phagemid capture. The standard column purification protocol from the Quick-DNA/RNA kit was then applied, and 15 μl of eluted ssDNA binders were stored at −20 °C for subsequent quantification or sequencing.

For the phage immunoprecipitation-based ligand discovery, 1 μg of EGFR-Fc (R&D Systems, Cat No. 344-ER) and Etanercept chimeric proteins (MilliporeSigma, Cat No. 185243–69–0), representing the extracellular domain of the target receptor fused to the Fc region of immunoglobulin G, was incubated with the preblocked phage library overnight at 4 °C. The following day, 20 μl of protein G beads (Thermo Fisher Scientific, Cat No. 10009D) were added to the phage-chimera mixture and rotated for 4 h at 4 °C to immunocapture all chimeras. The beads were then washed three times with PBS containing 0.01% NP-40 and stored in −80 °C before sequencing.

### NGS Sequencing and Data Analysis

Binders from both cell-based and bead-based screening methods were subjected to PCR for library insert amplification and sample-specific barcode incorporation. The PCR products were pooled and analyzed using Illumina sequencing. The protocol used here is a standard PhIP-Seq protocol that has been described in detail (33). Briefly, a first PCR was performed with primers that flank the displayed peptide inserts, and a subsequent PCR added adapters and sample indexes for single-end dual index Illumina sequencing.

Illumina sequencing FASTQ outputs were demultiplexed and mapped to the reference sequences by alignment. Perfect matches were counted to generate a read count matrix, with rows representing polypeptides in the library and columns corresponding to samples including the targets of interest and relevant negative controls. Each sample, including the negative control, was assayed in triplicate. For the cell-based screening method, the negative controls were parental, nontransduced cells. For the phage immunoprecipitation-based method, the negative controls were human isotype immunoglobulin G. EdgeR software (bioconductor.org/packages/release/bioc/html/edgeR.html) (34, 35) was adapted to calculate the maximum likelihood fold-changes and the *p*-values of differential abundance in order to assess the enrichment for each sample relative to the negative controls. We determined that a significantly enriched polypeptide (“hit”) should have a *p*-value less than 0.001, a fold-change of at least 5, and a read count of at least 15 in two of the triplicate samples (fold change values and *p* values were calculated with the EdgeR software). These criteria were established heuristically to achieve optimal sensitivity and reproducibility.

### Sequence-Based Network Graph Analysis and Multiple Sequence Alignment Evaluation

Network graph analyses were performed using R 4.2.0, with the network graphs generated through the R iGraph software package (cran.r-project.org). Enriched peptides for each receptor screened were chosen to create the corresponding network graphs based on their sequence homology. Connectivity between sequences was determined by aligning protein sequences with blastp, using the rBLAST package interfacing with the ncbi-blast+ (2.13.0) software (ebi.ac.uk/jdispatcher/sss/ncbiblast). Alignment parameters included "-evalue 1e-3 -max_hsps 1 -seg no -soft_masking false -word_size 5 -max_target_seqs 100,000 -comp_based_stats none". In the network graphs, polypeptides were represented by nodes, and those sharing sequence similarity while meeting alignment thresholds (E value < 0.001) were connected. The link width was proportional to the BLAST bit-scores. To explore potential conserved motifs, sequences from individual clusters with more than two peptides were extracted for multiple sequence alignment (MSA) using the Clustal Omega tool provided by the European Molecular Biology Laboratory's European Bioinformatics Institute (EMBL-EBI). The same sequences were also used for motif discovery using MEME Suite 5.4.1.

### Binding Assay for ERR1712142|105-166 and Human Structural Homologs Screening Against MRGPRX4

To detect and quantify the binding of the candidate ligands *via* flow cytometry, HEK293-MRGPRX4 and parental HEK293 cell lines were incubated with the tagged candidate ligands. Following incubation, the cells were stained with fluorescence-conjugated antibodies that target the tags for flow cytometric analysis. Cells were detached with cell dissociation buffer and collected after passing through 40 μm cell strainer. The collected cells were washed three times with ice-cold 1% PBSA. The cells were then incubated with ERR1712142 (fused with FLAG-tag), 30 nM TFPI (Acro Biosystems, Cat No. TFI-H5226, fused with His-tag), 30 nM SPINT2 (Sino Biological, Cat No. 10324-H08H, fused with His-tag) and 30 nM APP751 (a 751 amino-acid isoform of amyloid-beta precursor protein) (BioLegend, Cat No. 842601, fused with His-tag) prepared in 1% PBSA for 4 h at 4 °C on an end-to-end rotator. After washing the cells three times with ice-cold 1% PBSA, the cells were washed one more time with FACS washing buffer and then stained with Alexa Fluor 647-conjugated FLAG tag antibody (Thermo Fisher Scientific, Cat No. MA1-142-A647, 1:400 dilution) for ERR1712142 or His tag antibody (Thermo Fisher Scientific, Cat No. MA1-21315-A647, 1:1000 dilution) for human homologs for 10-15 min at room temperature in the dark. The cells were then washed three times with ice-cold FACS washing buffer and resuspended in 400 μl buffer for the following flow cytometry analysis. Flow cytometry analysis was conducted on Beckman Coulter CytoFLEX. Data from flow cytometry were processed on Flowjo software (www.flowjo.com).

### PRESTO-Tango Assay

The HTLA-MRGPRX4 and HTLA cells were seeded at a density of 1.3E+04 cells/well in a 96-well half-area flat-bottom opaque white plate that was pretreated with 0.1 mg/ml poly-D-lysine. The following day, cells were stimulated by addition of ursodeoxycholic acid (UDCA) at certain concentrations. For the modulator activity test, the cells were preincubated with TFPI, SPINT2, APP751, Osteoprotegerin (ACROBiosystems, Cat No. TNB-H5220), or aprotinin (Sigma-Aldrich, Cat No. A1153) for 1 h at 37 °C before addition of UDCA. The cells were then incubated for 12 h at 37 °C. After incubation, the medium was removed, and 25 μl per well Bright-Glo reagent (Promega Corporation, Cat No. E2620) was added. The plates were incubated for 5 min at room temperature in the dark. The luminescence was measured by FlexStation 3 system (Molecular Devices). The dose-response curves were generated using Graphpad Prism 9 software (www.graphpad.com).

### Lactate Dehydrogenase Cytotoxicity Assay

Cytotoxicity of proteins was measured by CyQUANT lactate dehydrogenase (LDH) cytotoxicity assay kit (Invitrogen, Cat No. C20300) following the manufacturer’s instructions. Briefly, the HTLA-MRGPRX4 and HTLA cells were seeded and treated by the same procedure as the PRESTO-tango assay. After treatment, 40 μl medium of each well was transferred to a 96-well flat-bottom plate for reactions. Additionally, cell lysis solution was added to untreated cells as lysed cell controls, and after complete lysis, 40 μl supernatant was transferred to the reaction plate. Subsequently, 40 μl per well reaction mixture was added to the reaction plate. The plates were incubated at room temperature for 30 min in the dark, and then 40 μl stop solution was added into each well. The absorbance at 490 nm and 680 nm was measured by Varioskan LUX Multimode Microplate Reader (Thermo Fisher Scientific). LDH activity was calculated by subtracting the 680 nm absorbance value from the 490 nm absorbance value. For calculation of % cytotoxicity, LDH activity of the untreated wells was subtracted from LDH activity of both treated wells and lysed cell controls; then LDH activity of treated wells was divided by activity of lysed cell controls.

### Docking Prediction

We used the AlphaFold-Multimer (36) implementation in ColabFold (github.com/sokrypton/ColabFold) (37) version 1.5.2, incorporating templates from PDB70, to perform docking of four targets on EGFR (epidermal growth factor (EGF), MIITX(02)-Mg1a, omega-HXTX-Hi2g_2, and phospholipase A2 homolog) and ERR1712142|105-166 on MRGPRX4. AlphaFold-Multimer generated five docked models for each target, of which we selected the top-ranked model, guided by the average pLDDT scores. We further subjected the top-ranked model to RosettaDock 4.0 (38), using conformational ensembles on ROSIE (39). From the 1000 models generated by RosettaDock, we selected the top 10 based on the Rosetta interface scores. To identify key amino acids involved in binding, we generated contact maps of the top-ranked models from both AlphaFold and RosettaDock using MAPIYA, considering the distance between amino acids with a cutoff set at 5 Å (40).

## Results

### Adaptation of Programmable M13 Hyperphage System to Ligand Discovery *via* Sequencing-Assisted Selection

To discover novel interactions involving AV peptide and venom-related peptide ligands with diverse structural characteristics, we first optimized a programmable M13 polyvalent “hyper” bacteriophage display screening platform (24). The M13 minor coat protein p3 is secreted through the *E. coli* periplasm where disulfide bond formation can occur, thus facilitating proper folding of fused venom-like polypeptides.

**Fig. 1:**
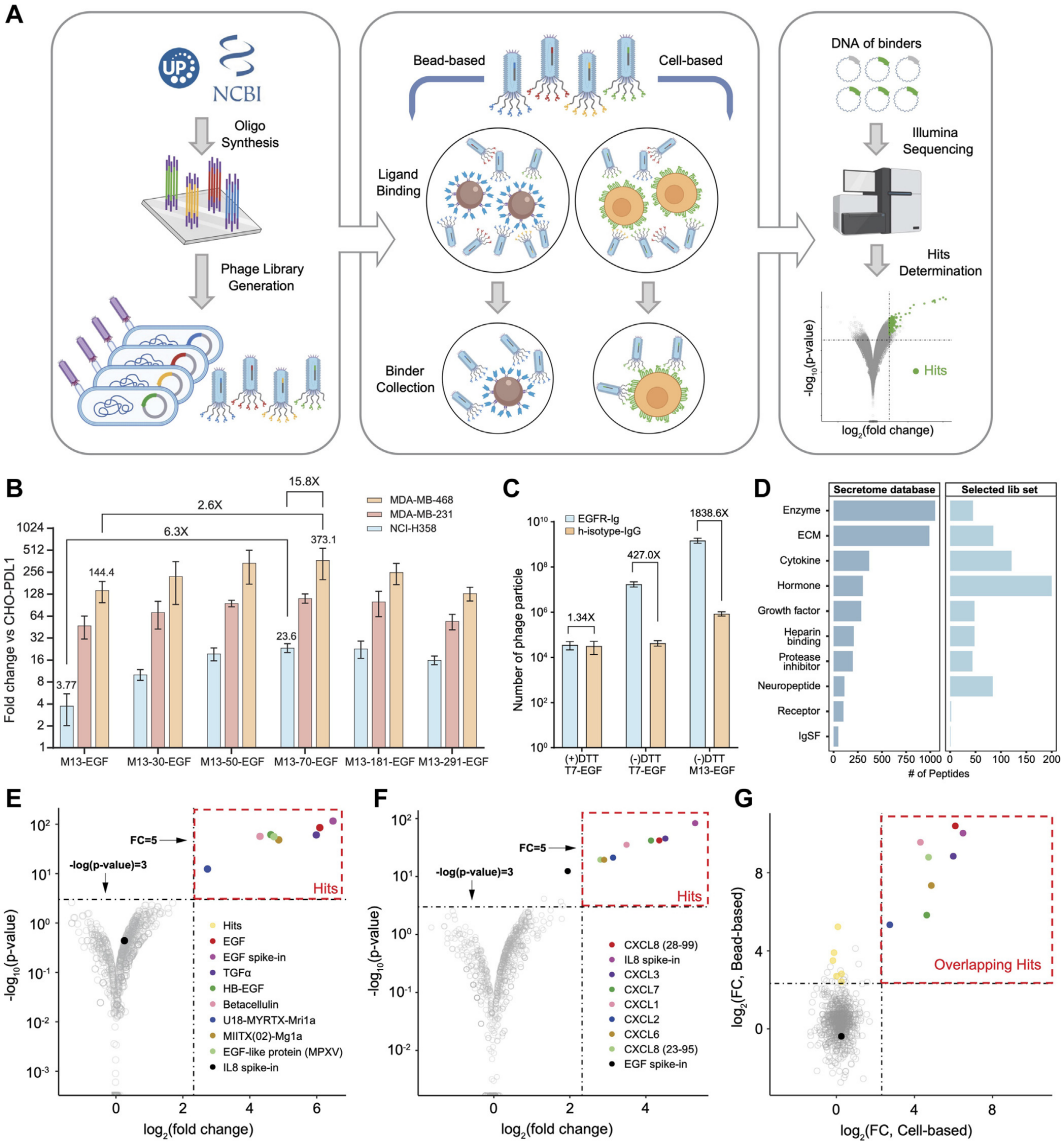
**M13 polyvalent phage display workflow identifies ligands for EGFR and CXCR2.***A*, a synthetic oligonucleotide library encoding full-length polypeptides up to 90 aa is designed and synthesized. The oligonucleotide library is cloned into the M13 hyperphage display system and grown up in *Escherichia coli* cells (*left*). Proteins of interest fused with the M13 P3 protein are displayed in five copies on the phage surface. The phage library is screened against a target (bead based or cell based) for affinity enrichment (*middle*). The DNA of the enriched phage library is collected and amplified by PCR for high-throughput sequencing. Informatic analysis identifies candidate binder peptides (*right*). *B*, the effect of linker length and cell surface receptor expression level on M13 phage displayed EGF enrichment. *C*, evaluation of disulfide bond formation and its impact on EGF binding to EGFR. *D*, the composition of the human secretome library by molecular function. *E* and *F*, volcano plot depicting the enrichment results of M13 secretome library screening against cells overexpressing EGFR (*E*) or CXCR2 (*F*). Each point is a unique secreted peptide. Known ligands are highlighted with distinct colors. The spiked-in control is colored *black*, and peptides not significantly enriched are colored *gray*. *G*, Scatter plot comparing peptide enrichment from EGFR-overexpressing cells (*X*-axis) *versus* Ig-EGFR coated magnetic beads (*Y*-axis). Each point is a unique secreted peptide from the library. Bead-only enriched peptides are colored *yellow*, while overlapping peptides and the spiked-in controls are colored as in (*E*). CXCR2, C-X-C motif chemokine receptor 2; ECM, extracellular matrix; EGFR, epidermal growth factor receptor; IgSF, immunoglobulin superfamily.

The discovery process consists of three main steps: phage library construction, single-round selection, and candidate identification *via* library sequencing-based analysis (Fig. 1*A*). We first developed and optimized our phage display vector and screening workflow by examining the interaction between EGF and native human EGFR (hEGFR). The EGF-hEGFR interaction is known to be high affinity (Kd = 0.1-1 nM) (41) and dependent on EGF’s three disulfide bonds. This well-characterized ligand-receptor pair therefore serves as an excellent positive control. To optimize binder selection efficiency, varying linker lengths were tested in the context of M13-displayed EGF binding to hEGFR expressed on the cell surface at varying densities. A range of linker lengths between 13 and 291 amino acids (Supplemental Table S1 and Supplemental Fig. S1*A*) were tested in conjunction with 3 cell lines of varying EGFR expression levels: high expression (MDA-MB-468), medium expression (MDA-MB-231), and low expression (NCI-H358). As a negative control, we used CHO K1 cells overexpressing programmed cell death ligand 1, which are known to have low levels of EGFR expression on their surface (Supplemental Fig. S1*B*).

The dependence of linker length was significantly more pronounced in the context of lower EGFR expression level. With a 70 aa-long linker, higher fold changes were observed across all cell lines, with approximately 16-fold higher enrichment between low (NCI-H358) and high (MDA-MB-468) EGFR expressing cells (Fig. 1*B*). A similar linker length-dependent trend was found when M13-displayed interleukin-8 was tested with cells overexpressing the interleukin-8 G-coupled protein receptor, C-X-C motif chemokine receptor 2 (Supplemental Fig. S1, *C* and *D*). Subsequent libraries were therefore constructed in a phagemid vector incorporating the 70 aa linker (M13–70).

The importance of the EGF disulfide bond formation was next evaluated by comparison of the M13 system with the T7 phage display system (Fig. 1*C*) in a bead-based Ig-EGFR binding assay (Fig. 1*A*). T7 phage are lytic and do not pass through the oxidizing periplasm during replication, thus forming disulfide bonds much less efficiently. EGF expressed on M13 demonstrated greatly increased binding relative to EGF expressed on T7. The lower level of T7-EGF binding was ablated by disulfide bond reduction with DTT, while T7 phage infectivity was unaffected.

### Rediscovery of Known Membrane Receptor Ligands with Programmable M13 Polyvalent Phage Display of the Human Secretome

To further assess how well the optimized M13 polyvalent display system could identify diverse ligands for cell surface expressed receptors, we constructed a library encompassing all 880 full-length human extracellular and secreted proteins whose mature polypeptide chains are equal to or less than 90 amino acids in length (the “human secretome library”). This library comprises approximately 66% of hormones listed in the Secretome database, including known EGFR ligands such as EGF, betacellulin (BTC), transforming growth factor-alpha (TGFα), and heparin-binding EGF-like growth factor (HB-EGF). We also incorporated three additional venom and venom-like proteins as positive controls for EGFR studies, namely, EGF-like peptide from monkeypox virus (MPXV), an EGF-like protein from the monkeypox virus, and two ant venom toxins, Mri1a and Mg1a, which are known EGFR agonists (42). In addition to hormones, nearly 33% of cytokines and 75% of neuropeptides from the comprehensive Secretome database are included in the human M13 secretome library. Most extracellular matrix proteins and secreted enzymes are not included in the library due to their sizes (Supplemental Table S2 and Fig. 1*D*). Despite this size limitation, the human M13 secretome library covers a diverse range of molecular functions, including hormones (200), enzymes (45), growth factors (48), protease inhibitors (44), and neuropeptides (84) (Supplemental Fig. S1*E*).

We employed both bead-based and cell-based panning approaches by incubating phage libraries with either magnetic beads coated with receptor extracellular domain-immunoglobulin fusion proteins or with cells overexpressing target receptors (Fig. 1*A*). Screens were performed in triplicate for statistical comparisons. Candidate ligands were identified by sequencing the recovered binders after washing away unbound phage particles (Fig. 1*A*). Many known endogenous EGFR ligands, including EGF, BTC, HB-EGF, TGFα, Mri1a, and Mg1a, were robustly identified using HEK293T cells overexpressing EGFR (Fig. 1*E*). Similarly, six of the seven known endogenous ligands of the seven-pass transmembrane GPCR CXCR2 (CXCL1, 2, 3, 5, 6, 7, and 8) were successfully detected by screening against CXCR2-overexpressing HEK293T cells (Fig. 1*F*). When panning against immunoglobulin-EGFR bound to magnetic beads, we identified a highly overlapping set of known and candidate ligands compared to cell-based screening, but with relatively increased fold-change values (Fig. 1*G*). These results establish good agreement between cell-based and bead-based screening methodologies, with enhanced signal strength and convenience for targets that can be presented at higher density on capture surfaces.

### Construction of a Comprehensive Animal Venom Scaffold Library

**Fig. 2:**
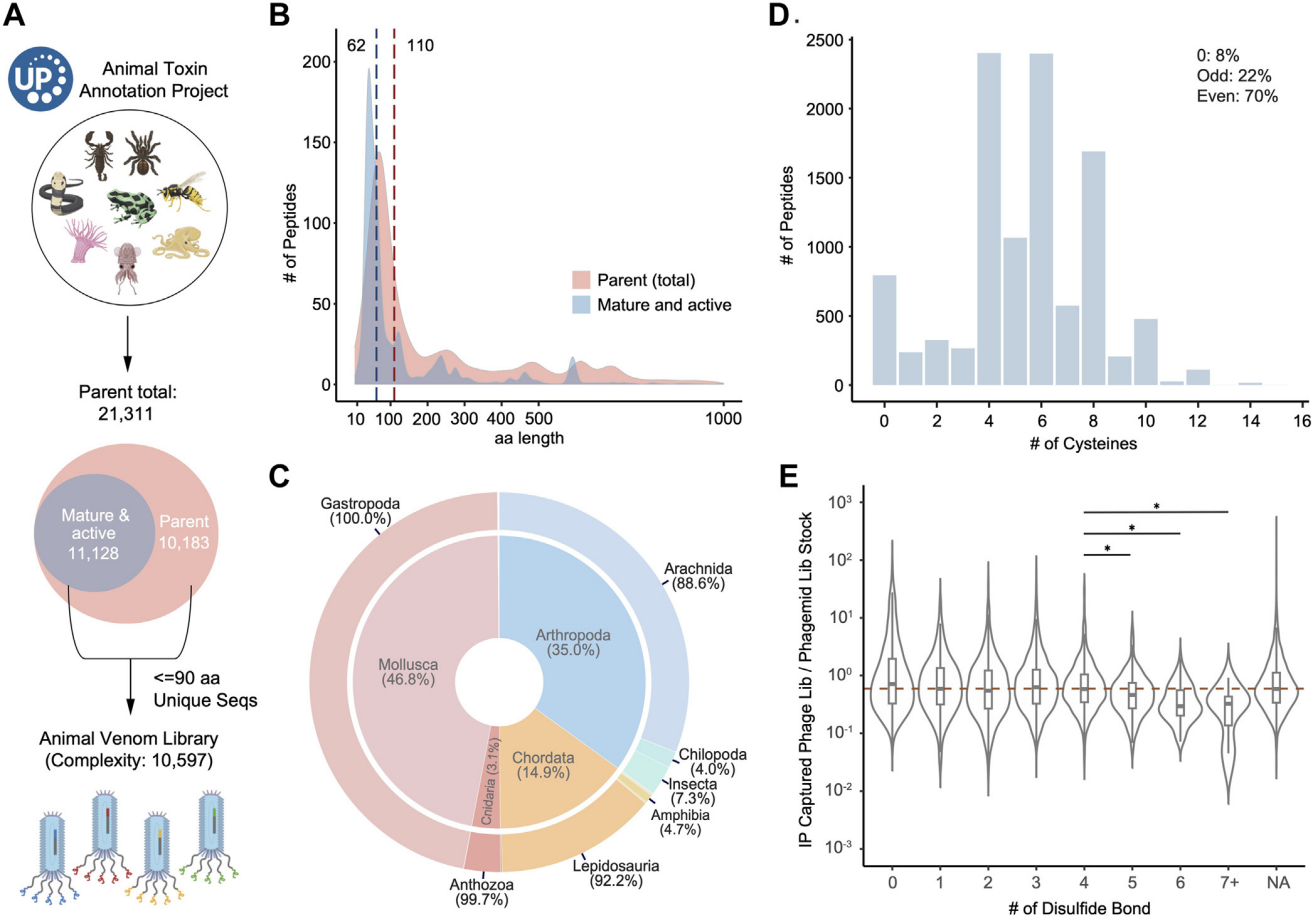
**Animal venom library design and characterization.***A* The 21,311 parent animal venom protein sequences were obtained from UniProt. Of these, 11,128 sequences possessing annotations for mature and active forms were extracted. Oligonucleotides encoding unique protein sequences up to 90 amino acids in length, regardless of their mature and active form status, comprise the M13 Animal Venom (AV) phage library. *B*, length distribution for AV sequences before and after the extraction of their mature and active forms. The median sequence length for the parent database is 110-aa, which reduces to 62-aa in their mature and active forms. *C*, composition of the AV library. The inner layer represents the animal phylum while the outer layer depicts the animal class. Supplemental Table S3 provides more detailed information. *D*, distribution of the number of cysteines in the AV library. *E*, relative abundance of the AV phage library members *versus* the phagemid library stock as a function of disulfide bonds described in UniProt. “NA” indicates sequences that possess cysteines but have unknown disulfide bond patterns. The *red dashed line* marks the median ratio (0.59) for the entire phage library. A two-tailed, unpaired *t* test with Welch's correction was used to assess the statistical significance of the decrease in ratio observed for library members with four or more disulfide bonds. A *p*-value of less than 0.05 was considered significant and is denoted by a *single asterisk*.

We next designed a diverse library covering all deposited animal protein venom sequences in the UniProt database, which encompasses highly divergent target classes and bioactivities. A total of 21,311 AV polypeptide sequences were retrieved from the UniProt database to serve as a parental set of sequences. By parsing annotations describing posttranslational modifications (PTMs) and/or processing events from UniProt, we were able to extract 11,128 mature and active forms of these sequences. The remaining 10,183 sequences lacked such features. The final AV library included 10,597 mature and active sequences that were less than or equal to 90 amino acids in length (49.7% of all parental sequences, Fig. 2, *A* and *B*).

The organisms represented in the final AV library span 22 taxonomic classes, with the majority of sequences originating from cone snails (Gastropoda), scorpions, and spiders (Arachnida), and snakes (Lepidosauria) (Fig. 2*C* and Supplemental Table S3). The library displayed a broad distribution of cysteine numbers: sequences devoid of cysteines make up 8% of the total, those with an odd number of cysteines account for 22%, while a substantial majority, 70%, contain an even number of cysteines. Remarkably, 90.2% sequences contain at least two cysteines, suggesting that disulfide bond formation is likely to be a key feature of this unique and diverse scaffold library (Fig. 2*D*).

In the cloned phagemid library, 99.6% of the designed AV sequences were detectable, with 98.2% of the sequences present within one log (plus or minus) of the median. After phage packaging, 90.8% of the sequences were observed in the final phage library (Supplemental Fig. S2, *A* and *B* and Supplemental Table S4), in accordance with the typical skewing and dropout of M13 phage display libraries. Factors contributing to skewness include uneven amplification, propagation, and secretion of phage library members. We observed no strong correlation between the yield of specific library members containing up to four disulfide bonds, above which a slight decrease in yield was associated with increasing cysteine residues (Fig. 2*E*). It is important to note that the number of disulfide bonds formed during expression of the fusion proteins produced in *E. coli* may differ from the annotation of the native polypeptide.

### Expansion of the AV Library *via* Sequence Homology Searching in Large Metagenomic Databases

**Fig. 3:**
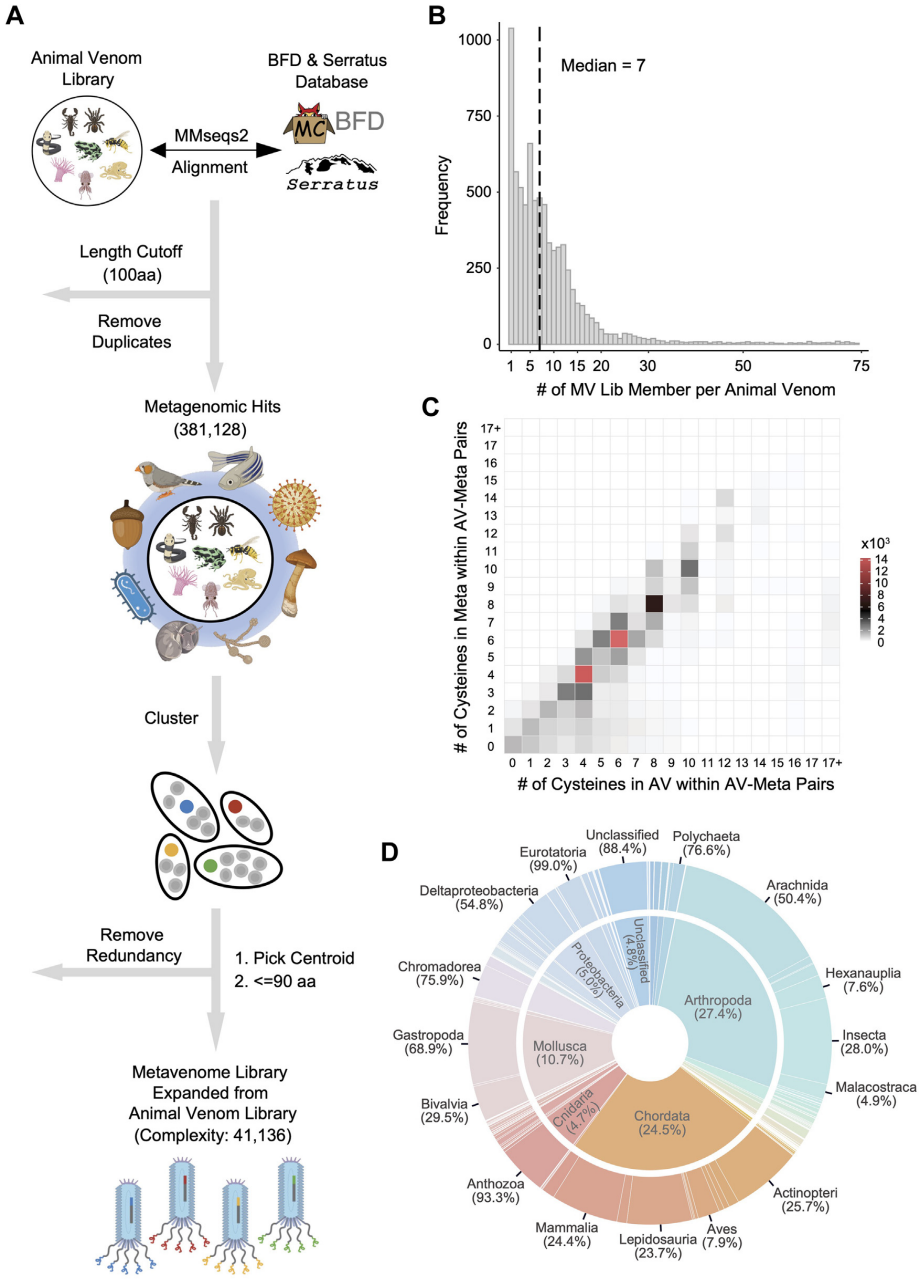
**Metavenome library design and characterization.***A*, animal venoms were used as annotated queries to search for homologous sequences in the Big Fantastic Database (BFD) and Serratus. Identical target sequences and those exceeding 100 amino acids were removed. Metavenome sequences were clustered and excluded if they showed at least 50% sequence identity and a 95% overlap with an animal venom sequence. The final metavenome library consisted of sequences of 90 amino acids or shorter, resulting in 41,136 unique sequences that were synthesized as the metavenome library DNA pool. These sequences were subsequently cloned and packaged to form the metavenome phage library. *B*, the number of metavenome sequences that corresponds to each animal venom. *C*, heat map depicting the correlation of the number of cysteines between animal venom and metavenome sequence pairs. *D*, composition of the metavenome library based on animal classification. The inner layer represents the animal phylum while the outer layer depicts the animal class. See Supplemental Table S6 for more detailed information.

To expand the diversity of our animal venom-related molecular scaffolds, we used the sequences of the AV library as queries to search for related sequence in the BFD and Serratus database using MMseqs2 (43) (Fig. 3*A*). We then removed any target sequences that were identical or longer than 100 amino acids resulting in 381,128 metagenomic sequences with venom-like attributes; referred to as the “metavenome” (Fig. 3*A*). To create a compact yet diverse library, we clustered the MV sequences at 50% sequence identity and 95% sequence length overlap using MMseqs2 and subsequently removed every sequence that overlaps with an existing AV sequence using the same thresholds (Fig. 3*A*); resulting in 41,136 representative sequences. The encoding sequences were synthesized as an oligonucleotide pool and cloned to create the M13 “metavenome” (MV) library (Fig. 3*A*).

We found a median value of seven metagenomic hits for each AV sequence (Fig. 3*B*) and importantly, a high correlation of cysteine numbers between AV and MV pairs (Fig. 3*C*). Whereas most other amino acids showed little or no correlation, phenylalanine and tyrosine did exhibit significant correlation, despite being less abundant (Supplemental Fig. S2, *C* and *D*). We observed no significant correlation between the yield of the phage particles and the number of cysteines in the MV protein sequences (Supplemental Fig. S2*E*). Briefly, 99.1% of the MV library members were successfully cloned into the M13-70 phagemid vector, and approximately 96% of the library members were represented in the final phage library (Supplemental Fig. S2, *A* and *B*).

Given the vast majority of sequences in the BFD and Serratus databases are not annotated, we used NCBI-BLAST+ to query the nonredundant (nr) database for protein annotations, including taxonomic classifications, that could be adapted to the MV library members. In addition, 96.3% of MV library members acquired an annotation in this way (Supplemental Fig. S2*F* and Supplemental Table S6), with a median e-value of 1.19e-15, suggesting a high level of confidence in the annotated results (Supplemental Fig. S2, *G*–*J*). The taxonomic diversity of the MV is vastly expanded compared to the AV library, comprising 130 different phyla and 210 different classes (Fig. 3*D* and Supplemental Table S6).

### Diverse EGFR Ligands Discovered from the AV and MV Libraries

**Fig. 4:**
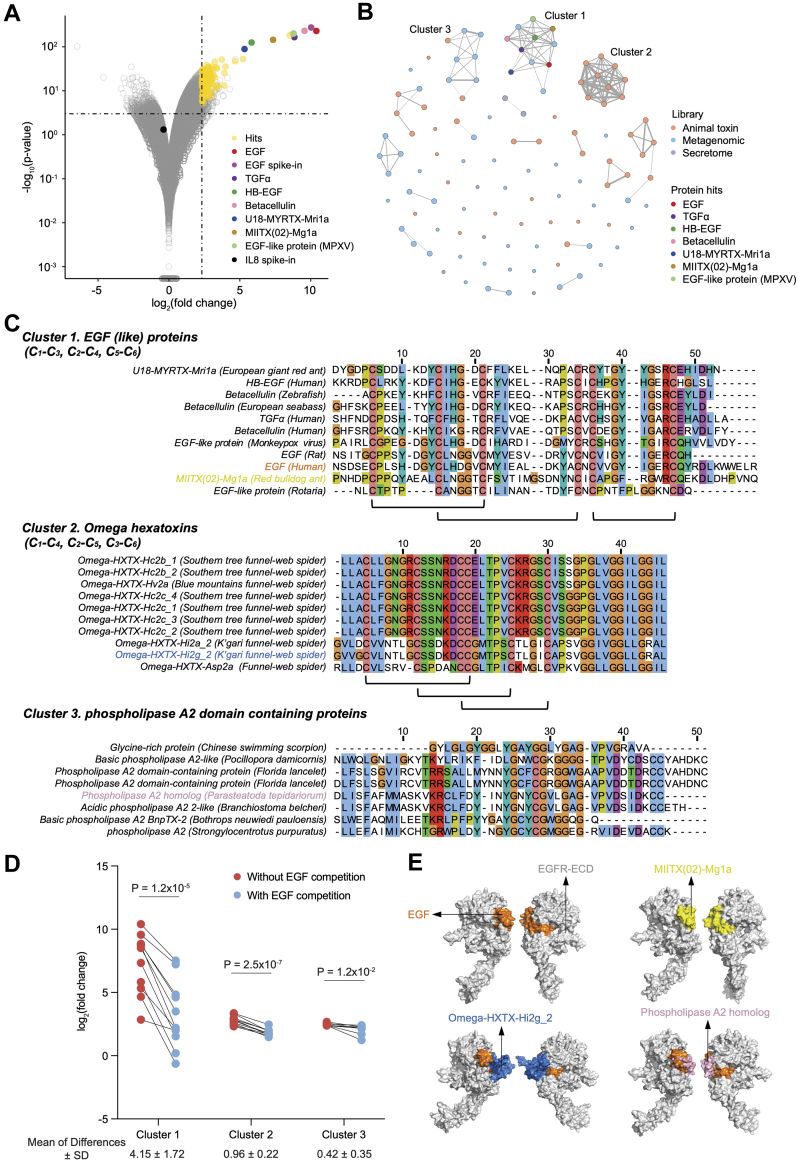
**Discovery of diverse scaffolds and ligands targeting EGFR from the secretome, AV, and MV libraries.***A*, volcano plots of secretome, AV, and MV libraries screened using bead-bound EGFR-Ig. Each point is a library member, with significantly enriched clones colored *yellow*. Endogenous and known ligands are color-coded as in Figure 1*E*. *B*, sequence-based network graph depicting sequence homologies among candidates. *Dot* color corresponds to the originating library. *C*, MSA analysis illustrates conserved amino acids shared among sequences within their respective clusters. *Black underlines* indicate the disulfide bond patterns provided in the UniProt database. *D*, the fold-change values represent ligands binding to EGFR in the presence and absence of EGF in clusters 1 to 3. The statistical significance of the fold-change values with and without EGF was assessed using two-tailed paired Student’s *t* tests. The mean differences of the fold-change values within the paired dots are displayed beneath each cluster. *E*, binding modes of selected ligands in each cluster predicted using RosettaDock. EGFR, epidermal growth factor receptor; MSA, multiple sequence alignment.

To evaluate the sensitivity of our platform to detect candidate binders with diverse affinities from a complex library, we again used the EGFR model system. We performed a comprehensive screening of the AV, MV, and secretome libraries using Ig-EGFR as the target. Candidate hits were determined by comparing sequencing results against a negative control immunoglobulin (Isotype Ig) from triplicate screens. We identified a multitude of candidate binders from all three libraries, in addition to the endogenous ligands (EGF, BTC, TGFα, and HB-EGF) and previously reported AV ligands (Mg1a and Mri1a) (Fig. 4*A*).

We used a sequence-based network graph approach to represent the sequence similarity among all candidate EGFR ligands. In this approach, candidate EGFR ligands, represented as nodes, were linked if they shared sequence similarity as determined by protein sequence alignment. Although many clones demonstrated no sequence homology to other hits, three dominant clusters emerged (Fig. 4*B*). To identify regions of sequence similarity that could underlie a shared binding mode, Clustal Omega was used to generate MSAs (Fig. 4*C*). Among all members of cluster 1, which includes human EGF, we observed absolute conservation of the cysteine amino acids known to be crucial for EGF binding. Cluster 2 was composed exclusively of sequences from spider omega hexatoxins of the AV library. Cluster 2 also exhibited absolute conservation of cysteine residues, but with a disulfide bond configuration distinct from EGF. Sequences in Cluster 3 did not appear to possess disulfide bonds, and instead appeared to feature a shared phospholipase A2 domain.

Conserved sequence motifs among candidate hits suggest a shared binding mode. Clusters 2 and 3 exhibited significantly lower binding strength to EGFR *versus* Cluster 1. In the presence of competing EGF, the binding strengths of ligands in Cluster 1 were greatly diminished, indicative of the expected competitive binding to the receptor (Fig. 4*D*, and Supplemental Fig. S3*A*). In contrast, EGF competition only minimally impacted binding of proteins in Clusters 2 and 3, suggestive of a distinct binding mode (Fig. 4*D*). To investigate this possibility, a representative from each cluster underwent RosettaDock modeling with EGFR (Fig. 4*E*). Ant venom peptide (MIITX(02)-Mg1a), a known activator of EGFR signaling, was selected from Cluster 1 and accurately docked into the ligand-receptor binding pocket. The strong binding ability of MIITX(02)-Mg1a, as well as its activation on EGFR, were further confirmed through an orthogonal flow cytometry-based binding assay and an EGFR activation assay (Supplemental Fig. S3, *B* and *C*). However, for the novel weaker binders from Cluster 2 and Cluster 3, their binding over background could not be confirmed in flow cytometry assessment, likely due to low binding affinity. Noticeably, from the docking result, Omega-HXTX-Hi2g_2 (Cluster 2) and Phospholipase A2 homolog (Cluster 3) docked outside of, but slightly overlapped with the ligand-receptor binding pocket (Fig. 4*E*), consistent with their insensitivity to EGF competition. This observation implies that these low-affinity candidate hits may have unique and interesting interactions with EGFR. While we could not detect antagonism in cell based assays, engineered derivatives of these lower-affinity binders may yield novel EGFR antagonists with clinical utility.

### Kunitz Type Domain Containing Proteins can Potentiate MRGPRX4 Signaling

To explore the broad utility of our approach for discovering novel venom-derived polypeptide binders, we sought to identify new ligands for the human itch receptor, MRGPRX4. The human MRGPRX4 receptor plays a crucial role in the perception of itch sensation (27, 28). Recent studies have illuminated that MRGPRX4 is activated by certain bile acids, such as UDCA. This activation triggers calcium-dependent neuronal excitation, culminating in the sensation of itch (27, 28). Activation of this pathway is believed to be a key feature of liver disease-associated cholestatic pruritus, marked by elevated levels of bile acids and other endogenous metabolites in the bloodstream. Modulation of MRGPRX4 activation is therefore being pursued as a promising treatment approach.

**Fig. 5:**
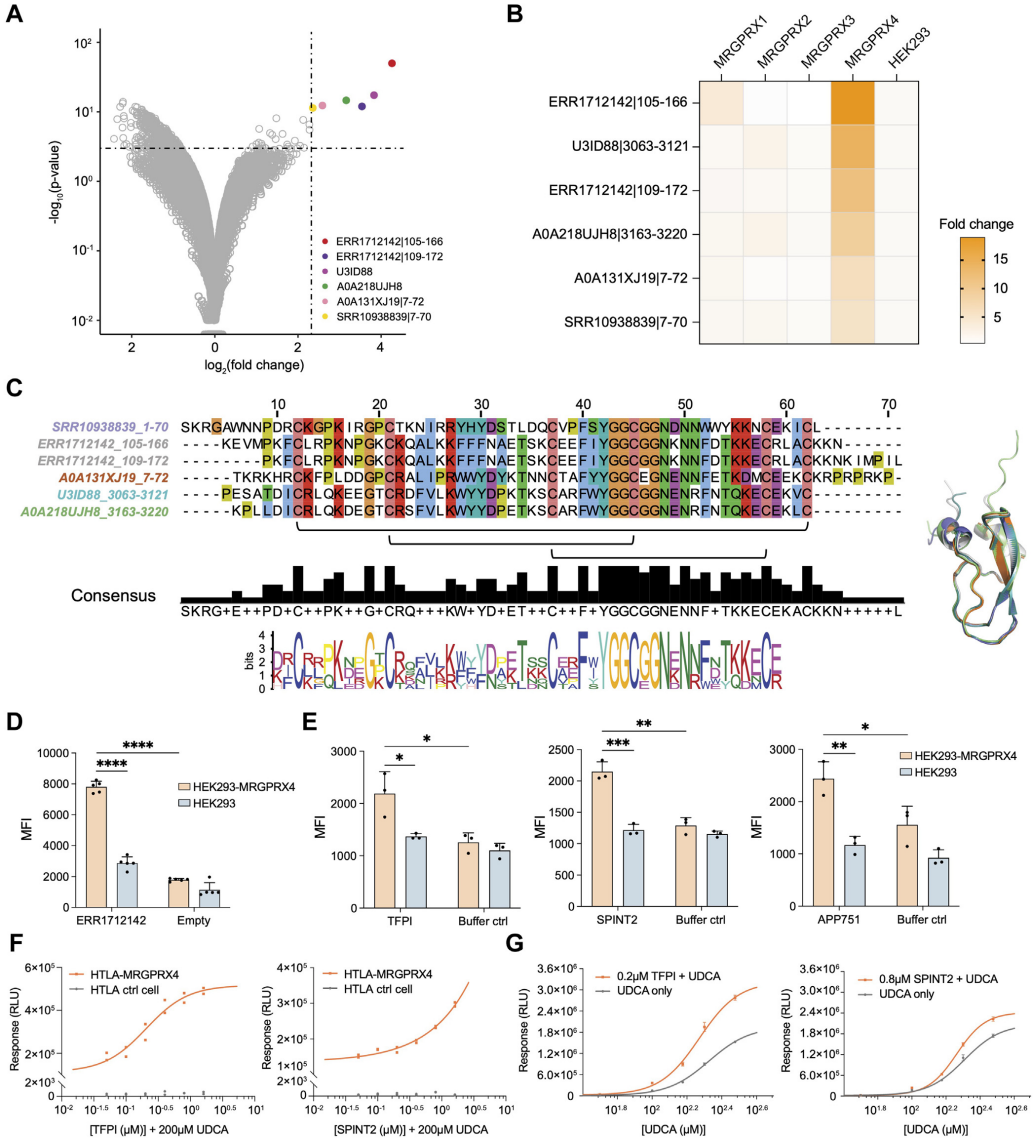
**Metavenom Kunitz type inhibitor domains can potentiate MRGPRX4.***A*, volcano plot of animal venom, metavenome, and secretome libraries binding to HEK293-MRGPRX4 cells *versus* HEK293 cells. Each point is a unique peptide in the AV, MV, and secretome libraries. *B*, heat map showing selectivity of fold-change values of the six peptide candidates across the HEK293-MRGPRX families. *C*, MSA analysis and motif discovery with MEME Suite identifies conserved amino acids shared among the candidate hits (*left*). Structural alignment of the hits using AlphaFold (*right*). *D*, flow cytometric analysis of ERR1712142|105-166 cell binding. MRGPRX4-overexpressing cells and parental HEK293 cells were incubated with ERR1712142|105-166 fused with a FLAG-tag. Following incubation, the cells were stained with Alexa Fluor 647-labeled anti-FLAG antibodies to quantify the amount of specifically bound candidate ligand *via* flow cytometry. An Empty control, which expressed irrelevant recombinant protein, was included as an additional negative control to ensure the specificity of the binding measurement. *E*, flow cytometric analysis of cell binding of TFPI (*left*), SPINT2 (*middle*), and APP751 (*right*). MRGPRX4-overexpressing cells and parental HEK293 cells were incubated with TFPI, SPINT2, or APP751 fused with a His-tag. Following incubation, the cells were stained with Alexa Fluor 647-labeled anti-His antibodies. MFI, median fluorescence intensity. Data are shown as mean ± SD of 5 (in *D*) or 3 (in *E*) biological replicates. Statistical comparisons use two-tailed unpaired Student’s *t* tests. ns, nonsignificant, ∗*p* < 0.05, ∗∗*p* < 0.01, ∗∗∗*p* < 0.001, ∗∗∗∗*p* < 0.0001. *F*, dose-response curves of TFPI (*left*) and SPINT2 (*right*) in the presence of 200 μM UDCA using the MRGPRX4 PRESTO-Tango assay. Each point is one replicate. RLU, relative luminescence unit. *G*, dose-responses curves of UDCA in the presence of 0.2 μM TFPI (*left*) or 0.8 μM SPINT2 (*right*) in the MRGPRX4 PRESTO-Tango assay. Data are shown as mean ± SD of three technical replicates. MSA, multiple sequence alignment; SPINT2, serine peptidase inhibitor, Kunitz type 2; TFPI, tissue factor pathway inhibitor; UDCA, ursodeoxycholic acid.

We conducted a screening campaign using our animal venom, metavenome, and secretome libraries using cells overexpressing MRGPRX4 (HEK293-MRGPRX4) and related members of the MRGPRX family (MRGPRX1, MRGPRX2, and MRGPRX3) (Fig. 5, *A* and *B*). Six proteins from the metavenome library demonstrated selective binding to MRGPRX4 (Fig. 5*B*). Interestingly, these proteins each share a highly conserved motif identifiable by MSA (Fig. 5*C*). High structural similarity among these six proteins is also predicted by AlphaFold (Fig. 5*C*).

To confirm binding of putative MRGPRX4 ligands, we produced the candidate hit with the highest fold-change value—ERR1712142|105-166—and evaluated binding to both MRGPRX4 overexpressing cells and parent HEK293 cells. ERR1712142|105-166 exhibited significantly higher binding to the MRGPRX4 overexpressing cells compared to control HEK293 cells (Fig. 5*D*).

We used Foldseek (44) to search for endogenous human homologs of these candidate MRGPRX4 binders. Foldseek interrogated AlphaFoldDB (version 4: Proteomes and Swiss-Prot), CATH clustered at 50% sequence identity, ESM Atlas-HQ, and the Protein Data Bank. All 20 of the unique human protein sequences identified as structural homologs to ERR1712142|105-166 are members of the Kunitz-type protease inhibitor class, which is characterized by six cysteines forming a distinctive disulfide bond pattern of C1-C6, C2-C4, and C3-C5 (Supplemental Fig. S4*A* and Supplemental Table S7). We therefore hypothesized that Kunitz-type inhibitor domain containing proteins might modulate MRGPRX4 signaling activity.

Among the human Kunitz-type inhibitor domain-containing proteins, TFPI, SPINT2, and an isoform of amyloid-beta precursor protein (APP751) demonstrated specific binding to MRGPRX4 overexpressing cells at 30 nM using a flow cytometry assay (Fig. 5*E*). These proteins are secreted but not included in the secretome library due to their sizes: TFPI with 254 amino acids, SPINT2 (extracellular domain) with 169 amino acids, and APP751 with 751 amino acids. The PRESTO-Tango assay is a luciferase-based method for measuring β-arrestin recruitment to activated GPCRs, and has been previously validated for detection of MRGPRX4 activation (45). We first conducted a cell cytotoxicity assay to ensure that prolonged exposure to these proteins would not induce cytotoxic effects (Supplemental Fig. S5*A*). Treatment of HTLA-MRGPRX4 cells with 0.4 μM TFPI and 1.6 μM SPINT2 showed no detectable cell cytotoxicity. However, APP751 exhibited toxicity at a concentration of 0.15 μM and was therefore excluded from subsequent testing (Supplemental Fig. S5*A*). The PRESTO-Tango assay revealed that neither TFPI nor SPINT2 activate MRGPRX4 independently (Supplemental Fig. S5*B*). However, TFPI and SPINT2 significantly potentiate MRGPRX4 activation (Supplemental Fig. S5*B*) in the presence of its agonist, UDCA. Dose responses of TFPI and SPINT2 were tested in the presence of UDCA at its half-maximal effective concentration (EC_50_) (Fig. 5*F*). As a potentiator, TFPI exhibited an EC_50_ of 0.2 μM, whereas SPINT2 showed a higher EC_50_ value, which was outside the dynamic range of the assay (Fig. 5*F*). Coincubation of UDCA with 0.2 μM TFPI or 0.8 μM SPINT2 greatly increased MRGPRX4 activation, but did not significantly shift the EC_50_ value of UDCA, consistent with activity most likely being mediated *via* positive allosteric modulation (Fig. 5*G* and Supplemental Fig. S5*C*). A negative control protein, osteoprotegerin, of comparable size to TFPI and manufactured under the same conditions, showed no bioactivity toward MRGPRX4 when incubated at TFPI’s EC_50_ concentration. A second negative control protein, aprotinin, a bovine Kunitz-type domain containing protein unrelated to TFPI or SPINT2, similarly demonstrated no bioactivity in the presence of UDCA, even at its maximally achievable concentration (Supplemental Fig. S5, *A* and *B*). Taken together, these observations demonstrate the potential of programmable phage display-based cell surface panning of animal venom and “metavenom” peptide libraries to discover bioactive lead compounds.

## Discussion

Animal venoms and related miniproteins exhibit unique structural and functional attributes including high potency, target selectivity, and serum stability. However, the potential of these molecules has remained largely underexploited, since the capability to screen diverse venom libraries in a molecular display format has not been developed. Conventional solutions for high-throughput screening have been based on bioactivity-guided fractionation, requiring large amounts of naturally sourced crude venoms (5, 17), or “sequence-based” approaches that have emerged alongside advances in transcriptomics and proteomics (5, 17). By leveraging existing venom polypeptide databases, several collections have been developed for use in high-throughput screening, but they cannot readily accommodate large library sizes (18, 19).

Here, we present an approach to construct and screen a comprehensive AV library, which was diversified to comprise the universe of sequences related to animal venoms: the “metavenome”. This fully synthetic, codon optimized, metavenome library, dramatically expands the search space of a screening campaign. Single-round M13 biopanning, combined with binder detection using high-throughput DNA sequencing, is shown to enable rapid discovery of lead molecular scaffolds spanning a range of binding strengths, overcoming the distortion, bottlenecking, and nonspecific binder selection effects that have limited the utility of traditional phage display workflows.

Biopanning against EGFR with the human secretome, AV, and MV libraries revealed known ligands and novel binders associated with diverse molecular scaffolds. MSA of the novel EGFR binders reveals conserved residues important for binding. Separately, panning in the presence and absence of excess EGF indicates whether an interaction is likely to be competitive *versus* allosteric. In the case of EGFR, for example, docking simulations predict that some of the novel binders we identified have the potential to interfere with EGF signaling if further optimized.

In an effort to discover novel therapeutic scaffolds relevant to cholestatic pruritus, we undertook a comprehensive screening campaign that identified six polypeptides that selectively bound to cell surface receptor MRGPRX4, each of which appeared to contain a Kunitz-type protease inhibitor domain. AlphaFold and RosettaDock predicted their mode of binding to MRGPRX4, which involved a 21-amino acid stretch of interacting residues (Supplemental Fig. S4*B*). In order to identify likely nonimmunogenic human protein lead candidates for therapeutic development, Foldseek found several Kunitz-type protease inhibitor domain-containing structural homologs, including TFPI, and SPINT2. In cell-based functional assays, both TFPI and SPINT2 demonstrated notable potentiation of MRGPRX4 activation in the presence of its endogenous ligand UDCA. We note that MRGPRX4, TFPI, and SPINT2 do not seem to have a significant tissue coexpression pattern that might otherwise suggest a physiologic role for these interactions.

While not demonstrated here, we propose that single-round, sequencing-assisted screening of venom and miniprotein libraries will be readily combined with machine learning approaches and generative artificial intelligence models to iteratively tailor library designs for specific targets or target classes (46, 47, 48). Biopanning in the presence or absence of competitive ligands, as demonstrated here, can also be useful for guiding candidate selection and iterative library design. Advanced structural modeling and docking algorithms, applied in conjunction with deep mutational scanning and other validation methods, will enable more informed scaffold engineering and lead candidate optimization. These strategies will significantly improve the speed and quality of lead generation. While a promising lead compound must still navigate the extensive downstream development stages and clinical trials, the efficient initial identification and optimization of potential drug candidates will accelerate and enhance the drug discovery and development pipeline.

It is important to recognize the limitations of this peptide drug discovery platform. The formation of disulfide bonds in *E. coli* cells may deviate from that in animal cells. This is important when considering mammalian homologs of novel binding candidates, and for downstream studies involving nonbacterial protein production systems. However, this limitation does not apply to all peptides in our libraries. In the AV library, 9.8% of library members have fewer than two cysteines, and the presence of disulfide bonds remains unknown for 53.3% of library members (Supplemental Table S4). Our metavenome library presents similar characteristics, with 12.8% of library members possessing fewer than two cysteines (Supplemental Table S5). It is also worth noting that for many peptides in the metavenome library, the species of the natural host were not annotated, further complicating the assessment of disulfide bond formation. The M13 phage display system produces libraries with significant distributional skewing. This effectively reduces the fraction of the library that is well-sampled during single-round screening campaigns. Eukaryotic display systems such as yeast, mammalian, or virus display could be alternatives to the M13 phage display system. Fully *in vitro* systems like the ParalleL Analysis of Translated ORFs (PLATO) (49) or Molecular Indexing of Proteins by Self Assembly (50) might also provide certain advantages, despite their monovalent display format. Importantly, the M13 phage display system is unable to produce proteins with most PTMs, which restricts the diversity of molecules that can be screened. Eukaryotic display systems present a promising alternative, as they enable the display of peptides with certain types of PTMs, including those required by the well characterized spider venoms that inhibit acid-sensing ion channels. Finally, as is true for any binding-based screening technologies, molecular display cannot be used for functional or phenotypic screening.

In summary, we have developed a peptide drug discovery platform which unites polyvalent M13 phage display of cysteine-rich animal venom-derived libraries, single-round sequencing-assisted selection, and deep sequence or structural modeling. We demonstrate the utility of the platform to discover ligands for both single and multipass membrane receptors expressed on intact human cells. Specifically, we identified known and novel EGFR binders, as well as endogenous potentiators of the itch sensor MRGPRX4. Further investigation of these molecular interactions may reveal a new strategy to block severe itch responses, such as in the setting of cholestatic pruritis. This drug discovery platform is therefore of broad utility for diverse classes of high value therapeutic targets.

## Data Availability

The data underlying this article will be shared on reasonable request to the corresponding author (hlarman1@jhmi.edu).

## Supplemental data

This article contains supplemental data.

## Conflict of interest

H. B. L., M. S., M. H., and Z. L. are listed as inventors on a patent application filed by Johns Hopkins University that covers the (meta)venom library design and construction, and M13 hyperphage ligand discovery platform. H. B. L. is a co-founder of Infinity Bio, Portal Bioscience, and Alchemab Therapeutics. The other authors declare that they have no conflicts of interest with the contents of this article
