# Supplementary material for: Molecular Display of the Animal Meta-Venome for Discovery of Novel Therapeutic Peptides
Source: Mol Cell Proteomics. 2024 Dec 31;24(2):100901. doi: 10.1016/j.mcpro.2024.100901 (PMC11833617; doi:10.1016/j.mcpro.2024.100901)
Supplement: Supplemental information [file mmc1.pdf]

Supplemental information for

Molecular Display of the Animal Meta-Venome for Discovery of Novel Therapeutic Peptides

Meng-Hsuan Hsiao[1,2,13], Yang Miao[1,3,13], Zixing Liu[1,4], Konstantin Schütze[5], Nathachit Limjunyawong[6,7], Daphne Chun-Che Chien[6], Wayne Denis Monteiro[1,2], Lee-Shin Chu[2], William Morgenlander[1], Sahana Jayaraman[1], Sung-eun Jang[5], Jeffrey J. Gray[2], Heng Zhu[9,10], Xinzhong Dong[6,8], Martin Steinegger[5,11,12,*], H. Benjamin Larman[1,*]

*Correspondence: hlarman1@jhmi.edu (H.B.L.), martin.steinegger@snu.ac.kr (M.S.)

This PDF file includes:

Supporting Information Text
Figures S1 to S5
Tables S1 to S7

Supporting Information Text

Supplemental details for Material and Methods

Animal venom, metavenome, and human secretome library design and synthesis

To obtain human secreted protein sequences, we accessed the UniProt database and downloaded entries based on the following search criteria: "taxonomy: 'Homo sapiens (Human) [9606]' (goa:('extracellular space [0005615]') OR goa:('extracellular region [5576]') OR locations:(location:'Secreted [SL-0243]'))". Subsequently, we extracted mature and active sequences with annotations or labels containing "chain" or "peptide" in the "PTM/Processing" section. For DNA synthesis, we retained only sequences that were equal to or less than 90 amino acids in length, regardless of whether they have mature and active forms. In total, 880 sequences were identified, and these were reverse translated with the pepsyn library design software. Sequences less than 270 base pairs were filled with PAS linker such that the final DNA length was brought up to 300 bases when appending primer binding sequences GGAATTCCGCTGCGT and CCGAGCATTGGCACC to the 5' and 3' end, respectively. This systematic approach for extracting mature and active sequences from the UniProt database was also applied in the design and generation of an animal venom library.

The animal venom library, comprising full-length active (mature) animal venom and poison protein sequences, was obtained from the UniProt animal toxin database using the search terms: taxonomy:"Metazoa [33208]" (keyword:toxin OR annotation:(type:"tissue specificity" venom)). To retrieve mature and active sequences, we employed the same method as described for the human secretome library generation. We extracted sequences both with and without mature and active forms and reverse translated 10,597 unique proteins that were equal to or shorter than 90 amino acids into their corresponding DNA using the pepsyn library design software. Sequences shorter than 270 bp were supplemented with PAS linkers, and primer binding sequences GGAATTCCGCTGCGT and GTCGTGCCAGGGAAC were appended to the 5' and 3' ends, respectively, to achieve a final DNA length of 300 bases.

To generate a metagenomic library expanding from the animal venom sequences, we first retrieved a list of known animal venoms. We searched the UniProt database for entries containing the keywords "toxin" and "animal". These toxins were used as queries for our searches against metagenomic databases. We searched for homologous sequences in two databases: the Big Fantastic Database (BFD) and a subset of SRA experiments identified by Serratus containing many RdRp's assembled by Plass(1). For the search, we used MMseqs2's iterative search with the following parameters: 3 iterations, high sensitivity, and a maximum of 1000 hits per sequence (--num-iterations 3 -a -s 7.5 --threads 100 --max-seqs 1000 --split-memory-limit 1T). The combined number of hits from both searches was 18,184,892. We then filtered these hits to include only proteins that cover the cleaved regions of the venome to at least 90%, reducing the set to 11,120,658. Using the alignment information and cleave annotations of the query, we inferred the cleaved regions of metagenomic sequences. This process yielded 4,424,507 cleaved sequences, with 1,283,667 being unique, across 9,388 queries. Next, we removed any identical (predicted) cleaved target sequences to reduce redundancy, resulting in 2,924,870 sequences, of which 739,724 are covering 8,202 queries. We also removed predicted cleaved regions longer than 100 amino acids, due to the size restriction of the phage display method, leaving 1,380,546 pairs, with 381,128 being unique, from 6,913 queries. Next we performed two steps to reduce the sequence set size while preserving diversity of our library: (1) we removed any metagenomic sequences with at least 50% sequence identity and a 95% overlap to a cleaved venom sequence by using MMseqs2's search; resulting in 333,787 unique pairs from 6,471 annotated queries. (2) We clustered the remaining cleaved sequences using MMseqs2 cluster(2) with a 95% overlap and 50% sequence identity (-c 0.95 --min-seq-id 0.5), which left 70,415 pairs from 5,424 queries. To counterbalance the venoms that were only paired with a few metagenomic sequences, we added back metagenomic sequences that were removed in the last step so that each venom had at least 5 metagenomic matches, resulting in the final set of 85,406 pairs from 5,941 annotated queries and 39,583 metagenomic hits. From the metagenomic hits, we further extracted 36,140 sequences that were equal to or shorter than 90 amino acids in length. To enhance sequence diversity, we included an additional 4,996 sequences without mature and active forms that

passed the same filtering process described above. In total, 41,136 sequences were reverse translated and supplemented with PAS linkers. To achieve a final DNA length of 300 bases, consistent with the human secretome and animal venom libraries, primer binding sequences GGAATTCCGCTGCGT and GCCTGGAGACGCCAC were appended to the 5' and 3' ends, respectively. The entire pipeline is implemented in python, which can be accessed at https://github.com/steineggerlab/phagedisplay-venoms. The code after the BFD/Serratus search stage runs in about 15min.

The sequences encoding the human secretome library, animal venom library, and the metagenomic library were all synthesized by Twist Bioscience (San Francisco, CA) and subsequently cloned into the M13-70 phagemid vector with EcoRI and HindIII restriction sites.

Protein production for ERR1712142 for binding assay

pLicC-MBP-ERR1712142 plasmid was extracted from bacterial lysate with Maxiprep (Qiagen Cat No. 12662) after overnight incubation in BL21 strain BL21 (λDE3) pLysS. The plasmids were transcribed in-vitro using the HiScribe T7 High Yield RNA Synthesis Kit (New England Biolabs Cat No. E2050S) to produce RNA. The 40 µL reaction contained 500 ng plasmid template, 20 µL NTP buffer mix and 4 µL T7 RNA polymerase and was incubated at 37 °C for 2 hours. After transcription the product was diluted with 60 µL molecular biology grade water DNA and the plasmid was cleaved at 37 °C for 15 minutes by the addition of DNAse I. Then 50 µL of 1 M LiCl was added to the solution and incubated at -20°C for 30 minutes. The centrifuge was cooled to 4°C, and the RNA was spun at maximum speed for 30 minutes. The supernatant was removed, and the RNA pellet washed with 70% ethanol. The sample was spun down at 4°C for another 10 minutes, and the 70% ethanol removed. The pellet was dried at room temperature for 15 minutes, and subsequently resuspended in 100 µL water.

RNA of translated using the PURExpress ΔRibosome Kit (New England Biolabs Cat No. E3313S). The translation reactions contained 0.4 µM mRNA, 10 µL Solution A, 3 µL Factor Mix, 0.3 µM Ribosomes, 20 U Murine RNase inhibitor (Protector RNase inhibitor, Millipore Sigma Cat No. 3335399001), 1 µL of Disulfide Bond Enhancer 1 and 1 µL of Disulfide Bond Enhancer 2 (New England Biolabs Cat No. E6820S). The reactions were incubated at 37 °C for 8 hours and used immediately or stored at -80 °C. 2.5 µL of the translated product was run on a 4-12% Bis-Tris SDS-PAGE gel and transferred onto a PVDF membrane and stained for validation of translation.

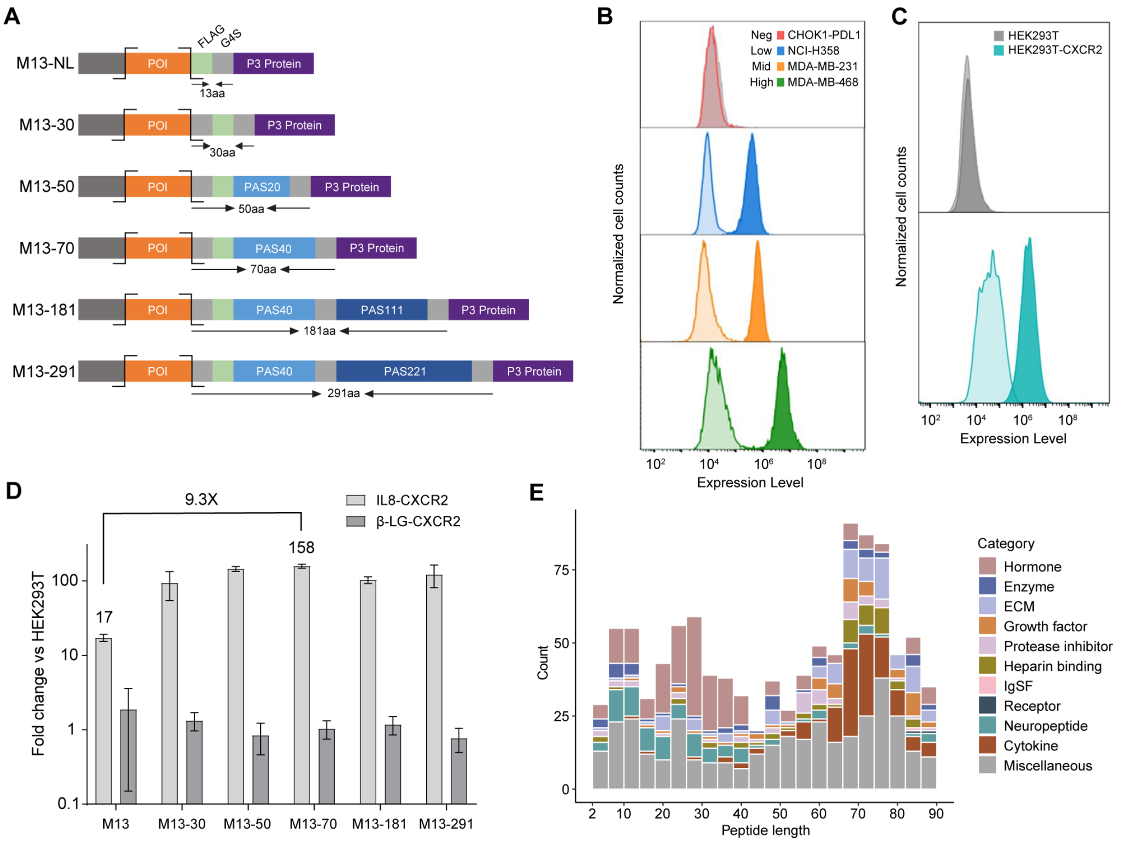

Fig. S1. M13 hyperphage display platform development and validation. (**A**) Depiction of the M13 phagemid vector with the key components labelled: POI, peptide of interest (orange), Flag tag (green), G4S linker (grey), PAS linkers (blue), P3 protein (purple), and restriction digestion sites (black lines). The linker lengths range from 13 (NL) to 291 amino acids. (**B**) Flow cytometry analysis of EGFR expression levels on four cell lines: CHOK1-PDL1 (negative control cell line), NCI-H358 (low), MDA-MB-231 (medium), and MDA-MB-468 (high). The lower expression level peaks represent background staining with only the fluorescent anti-mouse mAb, and the higher expression level peaks represent cells stained with anti-EGFR mAb (primary mAb) and detected with a fluorescent anti-mouse mAb. (**C**) Flow cytometry analysis of CXCR2 expression levels on HEK293T cells (CXCR2-) and HEK293T-CXCR2 (CXCR2+) cells. The lower expression level peaks represent background staining with only the fluorescent anti-mouse mAb, and the higher expression level peaks represent cells stained with anti-CXCR2 mAb (primary mAb) and detected with a fluorescent anti-mouse mAb. (**D**) Graph depicting the impact of linker length on the fold change value of IL-8 binding to CXCR2. β-Lactoglobulin (β-LG), serving as a negative control protein, exhibits low non-specific binding to HEK293T-CXCR2 cells. (**E**) Composition of the human secretome library. The histogram depicts the distribution of annotated protein molecular function versus amino acid length. The length of peptides ranges from 2 to 90 amino acids, split into 22 bins with width of 4 amino acids each bin.

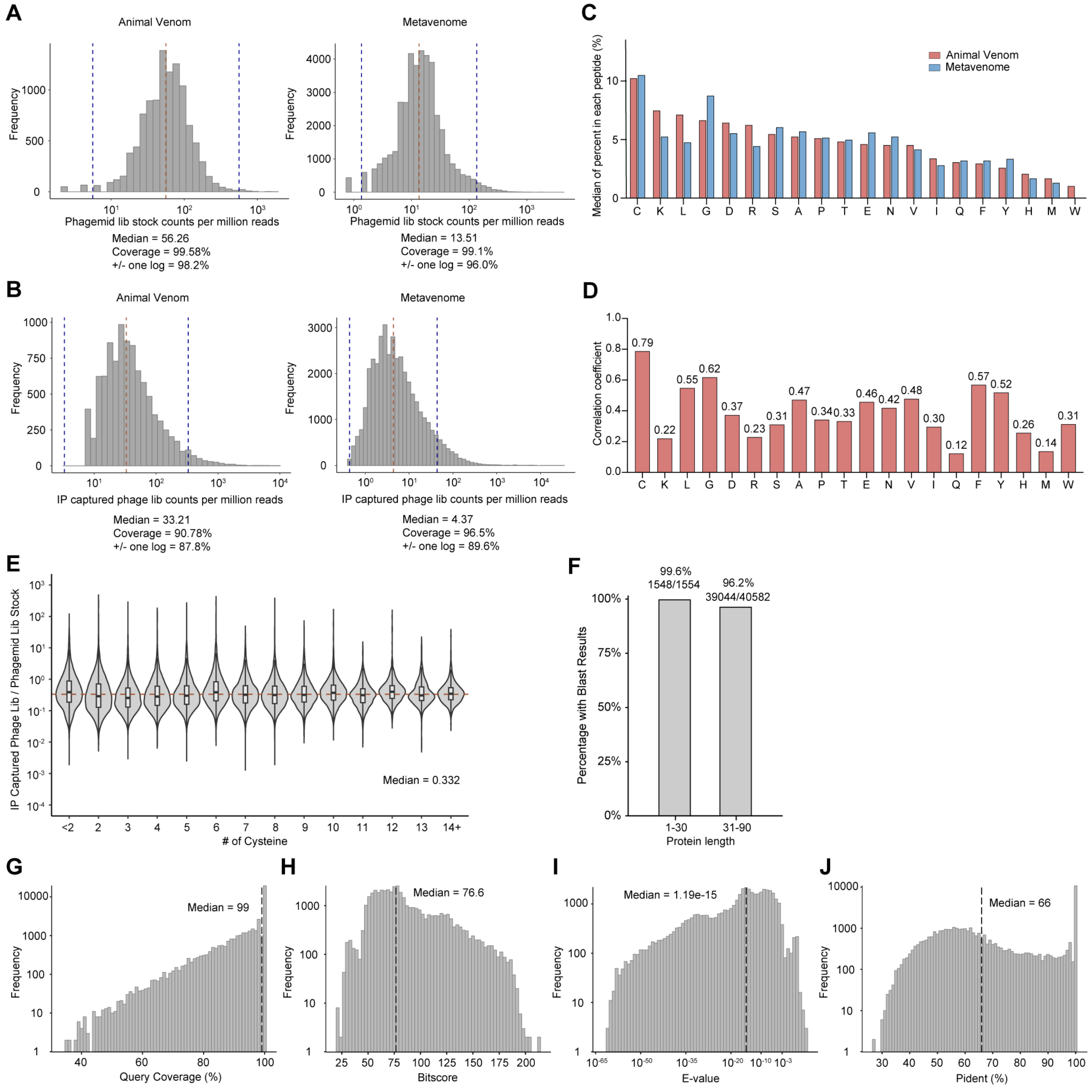

Fig. S2. Animal venom and metavenome library quality control and characterization. (**A**) Animal venom and Metavenome phagemid library stock quality control. For animal venom library, 99.58% of library members were detected; 98.2% of the library was within one log of the mean (indicated by vertical dashed lines). For metavenome library, 99.1% of library members were detected; 96.0% of the library was within one log of the mean. (**B**) Animal venom and Metavenome phage library quality control. For animal venom library, 90.78% of library members were detected; 87.8% of the library was within one log of the mean. For metavenome library, 96.5% of library members were detected; 89.6% of the library was within one log of the mean. (**C**) The abundance of each type of amino acid in the peptide sequences from both animal venom and metavenome libraries. Each set of bars represents a unique amino acid, with their heights corresponding to their relative abundance in the analyzed peptide sequences. (**D**) Analysis of amino acid abundance and correlation between animal venom and metavenome peptide sequence pairs. The Spearman correlation coefficient calculated from protein sequence pairs, with each bar corresponding to a distinct amino acid. The correlation coefficient is derived based on the quantity of each amino acid present in the animal venom and metavenome protein sequence pairs. (**E**) Assessment of the impact of the cysteine count on the metavenome phage library in comparison to its phagemid library stock, per number of expected cysteines. The same experimental method described for Figure 2E was used. (**F-J**) Metavenome library characterization with NCBI-BLAST+. (F) showed Library members annotated from NCBI-BLAST+. (G-J) showed essential parameters from NCBI-BLAST+ search outcomes with black dotted line in each plot highlighting the median value

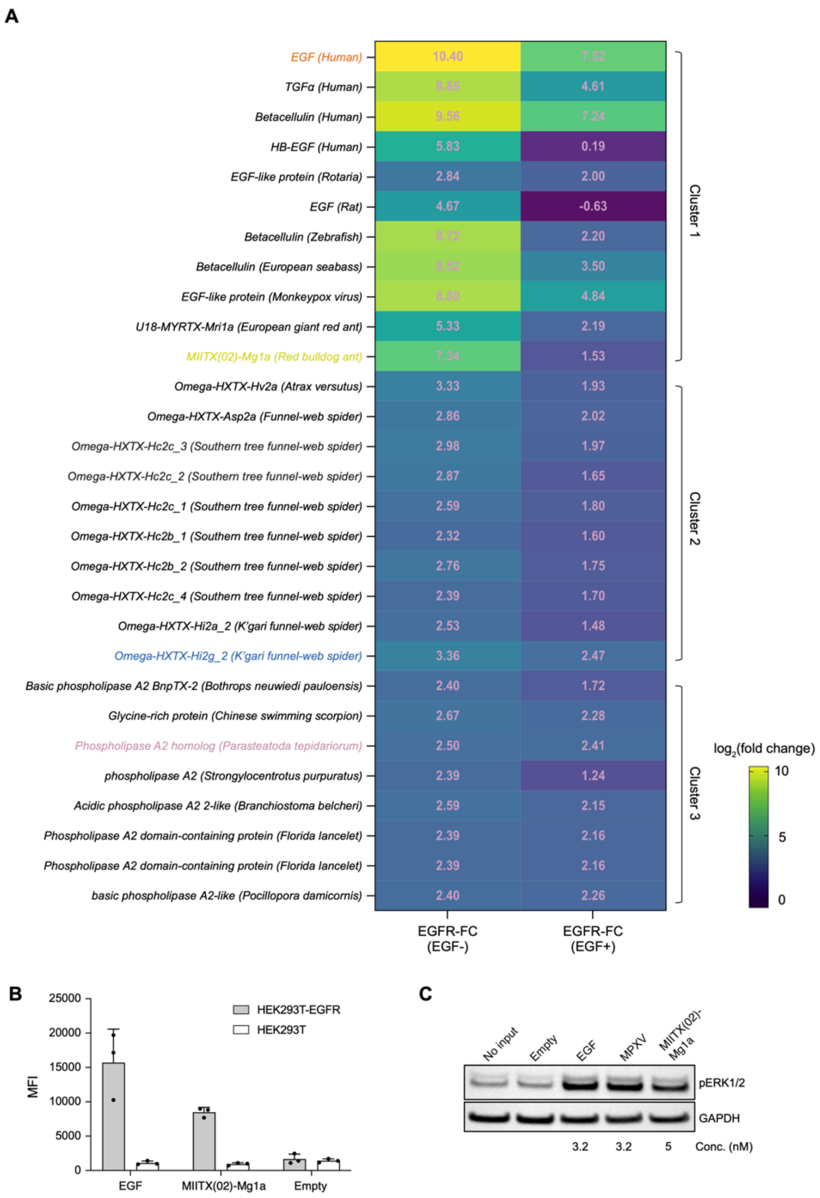

Fig. S3. Validation results for EGFR hits identified from screening. (A) Enrichment of EGFR ligands in cluster 1-3 from the animal venom and metavenome libraries. Fold change values for ligands binding to EGFR across clusters 1-3 are displayed in each heatmap cell. The left column presents screening results in the absence of EGF, while the right column depicts those obtained in the presence of competing EGF. **(B)** Flow cytometric analysis of MIITX(02)-Mg1a cell binding. EGFR-overexpressing cells and parental HEK293 cells were incubated with MIITX(02)-Mg1a fused with a FLAG-tag. Following incubation, the cells were stained with Alexa Fluor 647-labeled anti-FLAG antibodies to quantify the amount of specifically bound candidate ligand via flow cytometry. An Empty control, which expressed irrelevant recombinant protein, was included as an additional negative control to ensure the specificity of the binding measurement. MFI, median fluorescence intensity. Data are shown as mean ± SD of 3. **(C)** Anti-pERK1/2 western blot image demonstrating EGFR activation by recombinantly expressed protein. MDA-MB-468 cells with EGFR overexpression were incubated with 2 venom and venom-like hits (MIITX(02)-Mg1a and MPXV) at the indicated concentration for 30 minutes before harvest and downstream signaling activity analysis. MPXV, EGF-like protein form monkeypox virus. MIITX(02)-Mg1a, ant venom.

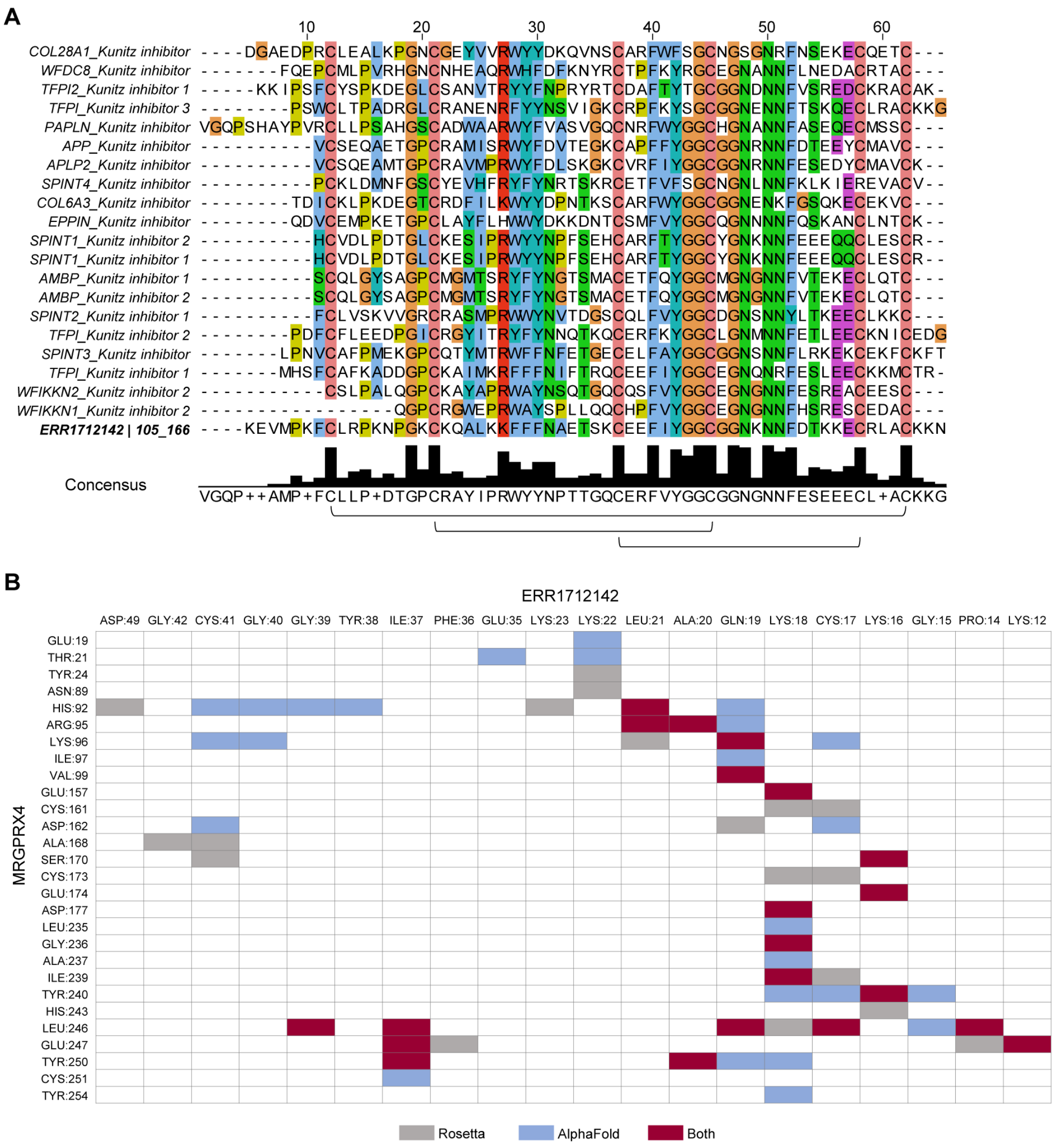

Fig. S4. Human structural Homologs to ERR1712142|105-166 identified with Foldseek and ERR1712142|105-166 docking result. (**A**) MSA analysis illustrates conserved amino acids shared between the 20 homologs and ERR1712142|105-166. Black underlines represent the disulfide bond patterns as annotated in the UniProt database. (**B**) Contact map of ERR1712142|105-166 -MRGPRX4 docking results via RosettaDock and AlphaFold. Amino acids between ERR1712142|105-166 and MRGPRX4 within a 5 Å radius are colored; consensus predictions between both modeling methods are indicated in red, while unique RosettaDock and AlphaFold predictions are displayed in grey and blue, respectively.

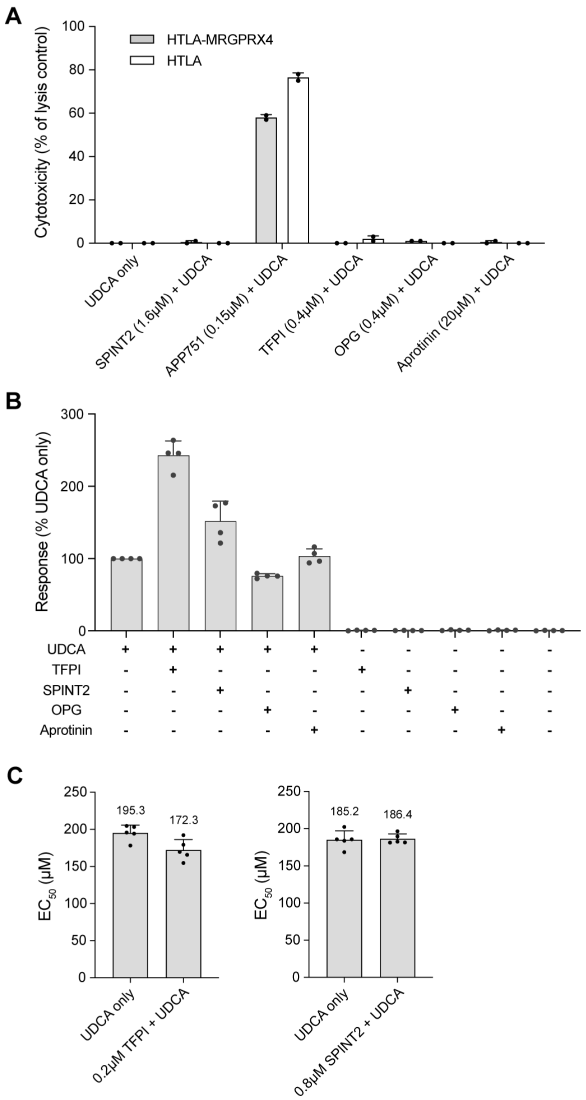

Fig. S5. Additional results for PRESTO-Tango and cytotoxicity assays. (**A**) Cytotoxicity of the proteins tested on PRESTO-Tango assay alongside UDCA on HTLA-MRGPRX4 and HTLA cells. Data are presented as the percentage relative to lysed cell control (mean ± SD, n=2). (**B**) Relative luminescence signal on HTLA-MRGPRX4 cells stimulated by 200μM UDCA, 0.2μM TFPI, 0.8μM SPINT2, and two negative control proteins, 0.4μM osteoprotegerin (OPG) and 20μM aprotinin on PRESTO-Tango assay. Data are presented as the percentage relative to UDCA only (mean ± SD, n=4). (**C**) EC_{50} of UDCA with or without 0.2μM TFPI or 0.8μM SPINT2 (mean ± SD, n=5).

Table S1. Sequences of tested M13 linker designs

Linker name	Short linker	FLAG_EK	PAS linker	GS linker	Addition PAS linker	Common
**M13-30 (30 aa)**	GGGGS	DYKDDDDK	X	(G4S)3	X	AS
**M13-50 (50aa)**	GGGGS	DYKDDDDK	**PAS20:** ASPAAPAPAS PAAPAPSAPA	(G4S)3	X	AS
**M13-70 (70aa)**	GGGGS	DYKDDDDK	**PAS40:** ASPAAPAPAS PAAPAPSAPA ASPAAPAPAS PAAPAPSAPA	(G4S)3	X	AS
**M13-181 (181aa)**	GGGGS	DYKDDDDK	**PAS40:** ASPAAPAPAS PAAPAPSAPA ASPAAPAPAS PAAPAPSAPA	(G4S)3	APR - [PAS38 - (G4S)3 - PS]1 - PAS38 - (G4S)3	AS
**M13-291 (291aa)**	GGGGS	DYKDDDDK	**PAS40:** ASPAAPAPAS PAAPAPSAPA ASPAAPAPAS PAAPAPSAPA	(G4S)3	APR - [PAS38 - (G4S)3 - PS]3 - PAS38 - (G4S)3	AS

Table S2. Composition of the human secretome library

	Secretome database		Human secretome lib	
	# of peptides	Proportion in original lib set (%)	# of peptides	Proportion in original lib set (%)
**Enzyme**	1050	16.38	45	5.10
**ECM**	991	15.46	85	9.64
**Cytokine**	368	5.74	121	13.72
**Hormone**	303	4.73	200	22.68
**Growth factor**	286	4.46	48	5.44
**Heparin binding**	209	3.26	48	5.44
**Protease inhibitor**	199	3.10	44	4.99
**Neuropeptide**	112	1.75	84	9.52
**Receptor**	102	1.59	2	0.23
**IgSF**	49	0.76	1	0.11
**Miscellaneous**	3523	54.96	372	42.18

Table S3. Animal venom library composition

Phylum	Class	# of lib members
Mollusca (46.83%)	Gastropoda	4962
	Cephalopoda	1
Arthropoda (34.98%)	Arachnida	3285
	Insecta	272
	Chilopoda	148
	Branchiopoda	1
	Merostomata	1
Chordata (14.92%)	Lepidosauria	1458
	Amphibia	74
	Actinopteri	20
	Mammalia	15
	Aves	12
	Chondrichthyes	2
Cnidaria (3.11%)	Anthozoa	329
	Scyphozoa	1
Platyhelminthes (0.06%)	Rhabditophora	3
	Trematoda	2
	Cestoda	1
Nemertea (0.04%)	Pilidiophora	4
Annelida (0.03%)	Polychaeta	3
Porifera (0.02%)	Demospongiae	2
Nematoda (0.01%)	Enoplea	1

Table S4. Animal venom library quality analysis

	Animal toxin lib db	Phagemid library stock		IP captured phage lib	
# of Disulfide bonds	# of peptides	# of peptides	Coverage	# of peptides	Coverage
0	1033	1019	98.6%	931	90.1%
1	210	209	99.5%	193	91.9%
2	435	434	99.8%	402	92.4%
3	1756	1754	99.9%	1583	90.1%
4	1137	1131	99.5%	1013	89.1%
5	243	242	99.6%	206	84.8%
6	121	121	100.0%	95	78.5%
7	13	13	100.0%	12	92.3%
8	2	2	100.0%	1	50.0%
10	1	1	100.0%	1	100.0%
Unknown (NA)	5646	5626	99.6%	5183	91.8%
Total	10597	10552	99.6%	9620	90.8%

Table S5. Metavenome library quality analysis

	Metavenome lib db	Phagemid library stock		IP captured phage lib	
# of Cysteine	# of peptides	# of peptides	Coverage	# of peptides	Coverage
0	2806	2754	98.1%	2693	96.0%
1	2472	2447	99.0%	2379	96.2%
2	2722	2711	99.6%	2621	96.3%
3	3618	3602	99.6%	3424	94.6%
4	2228	2200	98.7%	2121	95.2%
5	3440	3409	99.1%	3306	96.1%
6	9704	9596	98.9%	9433	97.2%
7	3223	3197	99.2%	3114	96.6%
8	4406	4378	99.4%	4233	96.1%
9	1690	1674	99.1%	1634	96.7%
10	2130	2114	99.2%	2087	98.0%
11	813	809	99.5%	791	97.3%
12	793	786	99.1%	783	98.7%
13	356	353	99.2%	351	98.6%
14	476	476	100.0%	476	100.0%
15	188	187	99.5%	186	98.9%
16	61	60	98.4%	59	96.7%
17	5	5	100.0%	5	100.0%
19	5	5	100.0%	5	100.0%
Total	41136	40763	99.1%	39701	96.5%

Table S6. Metavenome library composition: top 10 phyla

Phylum	Class	# of lib members
Arthropoda (27.43%)	Arachnida	5826
	Insecta	3241
	Hexanauplia	877
	Malacostraca	569
	Collembola	460
	Branchiopoda	275
	Ostracoda	97
	Merostomata	76
	Thecostraca	70
	Chilopoda	42
	Pycnogonida	20
	Diplopoda	1
	None	1
	Symphyla	1
Chordata (24.46%)	Actinopteri	2653
	Mammalia	2514
	Lepidosauria	2445
	Aves	811
	None	494
	Amphibia	381
	Leptocardii	370
	Appendicularia	210
	Ascidiacea	199
	Chondrichthyes	124
	Hyperoartia	60
	Cladistia	45
	Myxini	1
Mollusca (10.71%)	Gastropoda	3108
	Bivalvia	1329
	Cephalopoda	75
Proteobacteria (5.03%)	Deltaproteobacteria	1163
	Gammaproteobacteria	370
	Alphaproteobacteria	275
	Betaproteobacteria	203
	None	70
	Epsilonproteobacteria	19
	Oligoflexia	16
	Hydrogenophilalia	3
	Acidithiobacillia	1
	Candidatus Muproteobacteria	1
Cnidaria (4.74%)	Anthozoa	1864
	Hydrozoa	109
	Scyphozoa	16
	Myxozoa	6
	Cubozoa	2
Nematoda (3.76%)	Chromadorea	1202
	Enoplea	381

Table S6. (continued)

Phylum	Class	# of lib members
Rotifera (2.21%)	Eurotatoria	924
	Pararotatoria	9
Platyhelminthes (1.68%)	Cestoda	403
	Trematoda	214
	Rhabditophora	83
	Monogenea	9
Ascomycota (1.68%)	Sordariomycetes	200
	Eurotiomycetes	159
	Dothideomycetes	120
	Leotiomycetes	117
	Pezizomycetes	50
	Lecanoromycetes	31
	Geoglossomycetes	8
	Saccharomycetes	8
	Orbiliomycetes	5
	Pneumocystidomycetes	4
	Neolectomycetes	2
	Schizosaccharomycetes	1
	Taphrinomycetes	1
Annelida (1.37%)	Polychaeta	441
	Clitellata	135

Table S7. Structural Homologs to ERR1712142|105-166 in Human Proteins Identified with Foldseek.

Target ID	uniprot_ID	Seq.id.	Query Pos_start	Query Pos_end	Target Pos_start	Target Pos_end	E-Value	Score	Query seq.	Target seq.	Protein	Domain	Gene
AF-P05067-F1-model_v4	P05067	40.3	7	58	290	341	1.71E-05	257	FCLRPKNPGKCKQALKKFFFNAETSKCEEFIYGGCGGNKNNFDTKKECRLAC	VCSEQAETGPCRAMISRWYFDVTEGKCAPFFYGGCGGNRNNFDTEEYCMAVC	Amyloid-beta precursor protein	BPTI/Kunitz inhibitor	APP (A4, AD1)
AF-Q06481-F1-model_v4	Q06481	39.6	7	59	309	361	1.71E-05	258	FCLRPKNPGKCKQALKKFFFNAETSKCEEFIYGGCGGNKNNFDTKKECRLACK	VCSQEAMTGPCRAVMPRWYFDLSKGKCVRFIYGGCGGNRNNFESEDYCMAVCK	Amyloid-like protein 2	BPTI/Kunitz inhibitor	APLP2
AF-Q8TEU8-F1-model_v4	Q8TEU8	39.2	8	58	386	436	3.40E-04	208	CLRPKNPGKCKQALKKFFFNAETSKCEEFIYGGCGGNKNNFDTKKECRLAC	CSLPALQGPCKAYAPRWAYNSQTGQCQSFVYGGCEGNGNNFESREACEESC	WAP, Kazal, immunoglobulin, Kunitz and NTR domain-containing protein 2	BPTI/Kunitz inhibitor 2	WFIKKN2
AF-P49223-F1-model_v4	P49223	46.5	4	61	32	89	4.08E-06	286	MPKFCLRPKNPGKCKQALKKFFFNAETSKCEEFIYGGCGGNKNNFDTKKECRLACKKN	LPNVCAFPMEKGPCQTYMTRWFFNFETGECELFAYGGCGGNSNNFLRKEKCEKFCKFT	Kunitz-type protease inhibitor 3	BPTI/Kunitz inhibitor	SPINT3
AF-P48307-F1-model_v4	P48307	40.6	2	60	152	210	1.1E-06	304	EVMPKFCLRPKNPGKCKQALKKFFFNAETSKCEEFIYGGCGGNKNNFDTKKECRLACKK	KKIPSFCYSPKDEGLCSANVTRYYFNPRYRTCDAFTYTGCGGNDNNFVSREDCKRACAK	Tissue factor pathway inhibitor 2	BPTI/Kunitz inhibitor 1	TFPI2
AF-O43291-F1-model_v4	O43291	42.3	7	58	37	88	2.97E-05	269	FCLRPKNPGKCKQALKKFFFNAETSKCEEFIYGGCGGNKNNFDTKKECRLAC	FCLVSKVVGRCRASMPRWWYNVTDGSCQLFVYGGCDGNSNNYLTKEECLKKC	Kunitz-type protease inhibitor 2	BPTI/Kunitz inhibitor 1	SPINT2
AF-P10646-F1-model_v4	P10646	49.1	4	60	50	106	1.60E-06	301	MPKFCLRPKNPGKCKQALKKFFFNAETSKCEEFIYGGCGGNKNNFDTKKECRLACKK	MHSFCAFKADDGPCKAIMKRFFFNIFTRQCEEFIYGGCEGNQNRFESLEECKKMCTR	Tissue factor pathway inhibitor	BPTI/Kunitz inhibitor 1	TFPI
1tfx_D	P10646	43.8	5	61	2	58	1.61E-06	283	PKFCLRPKNPGKCKQALKKFFFNAETSKCEEFIYGGCGGNKNNFDTKKECRLACKKN	PDFCFLEEDPGICRGYITRYFYNNQTKQCERFKYGGCLGNMNNFETLEECKNICEDG	Tissue factor pathway inhibitor	BPTI/Kunitz inhibitor 2	TFPI
1irh_A	P10646	47.3	5	61	5	61	2.22E-04	187	PKFCLRPKNPGKCKQALKKFFFNAETSKCEEFIYGGCGGNKNNFDTKKECRLACKKN	PSWCLTPADRGLCRANENRFYYNSVIGKCRPFKYSGCGGNENNFTSKQECLRACKKG	Tissue factor pathway inhibitor	BPTI/Kunitz inhibitor 3	TFPI
AF-P02760-F1-model_v4	P02760	42.3	7	58	230	281	3.39E-05	246	FCLRPKNPGKCKQALKKFFFNAETSKCEEFIYGGCGGNKNNFDTKKECRLAC	SCQLGYSAGPCMGMTSRYFYNGTSMACETFQYGGCMGNGNNFVTEKECLQTC	Protein AMBP	BPTI/Kunitz inhibitor 1	AMBP
AF-O95925-F1-model_v4	O95925	36.3	5	59	74	128	8.64E-05	231	PKFCLRPKNPGKCKQALKKFFFNAETSKCEEFIYGGCGGNKNNFDTKKECRLACK	QDVCEMPKETGPCLAYFLHWWYDKKDNTCSMFVYGGCQGNNNNFQSKANCLNTCK	Eppin	BPTI/Kunitz inhibitor	EPPIN
AF-Q96NZ8-F1-model_v4	Q96NZ8	35.5	14	58	365	409	0.003016	166	PGKCKQALKKFFFNAETSKCEEFIYGGCGGNKNNFDTKKECRLAC	QGPCRGWEPRWAYSPLLQQCHPFVYGGCEGNGNNFHSRESCEDAC	WAP, Kazal, immunoglobulin, Kunitz and NTR domain-containing protein 1	BPTI/Kunitz inhibitor 2	WFIKKN1

Table S7. (continued)

Target ID	uniprot_ID	Seq.id.	Query Pos_start	Query Pos_end	Target Pos_start	Target Pos_end	E-Value	Score	Query seq.	Target seq.	Protein	Domain	Gene
AF-Q8IUA0-F1-model_v4	Q8IUA0	34.5	4	58	91	145	4.09E-05	232	MPKFCLRPKNPGKCKQALKKFFFNAETSKCEEFIYGGCGGNKNNFDTKKECRLAC	FQEPCMLPVRHGNCNHEAQRWHFDFKNYRCTPFKYRGCEGNANNFLNEDACRTAC	WAP four-disulfide core domain protein 8	BPTI/Kunitz inhibitor	WFDC8
AF-Q2UY09-F1-model_v4	Q2UY09	27.5	1	58	1065	1122	3.19E-05	229	KEVMPKFCLRPKNPGKCKQALKKFFFNAETSKCEEFIYGGCGGNKNNFDTKKECRLAC	DGAEDPRCLEALKPGNCGEYVVRWYYDKQVNSCARFWFSGCNGSGNRFNSEKECQETC	Collagen alpha-1(XXVIII) chain	BPTI/Kunitz inhibitor	COL28A1
AF-O43278-F1-model_v4	O43278	35.8	7	59	390	442	9.79E-05	231	FCLRPKNPGKCKQALKKFFFNAETSKCEEFIYGGCGGNKNNFDTKKECRLACK	HCVDLPDTGLCKESIPRWYYNPFSEHCARFTYGGCYGNKNNFEEEQQCLESCR	Kunitz-type protease inhibitor 1	BPTI/Kunitz inhibitor 2	SPINT1
AF-Q6UDR6-F1-model_v4 Kun	Q6UDR6	37.7	7	59	40	92	1.33E-04	238	FCLRPKNPGKCKQALKKFFFNAETSKCEEFIYGGCGGNKNNFDTKKECRLACK	PCKLDMNFGSCYEVHFRYFYNRTSKRCETFVFSGCNGNLNNFKLKIEREVACV	Kunitz-type protease inhibitor 4	BPTI/Kunitz inhibitor	SPINT4
AF-O95428-F1-model_v4	O95428	35.4	2	58	743	804	1.53E-02	134	EVMPKF-----CLRPKNPGKCKQALKKFFFNAETSKCEEFIYGGCGGNKNNFDTKKECRLAC	VGQPSHAYPVRCLLPSAHGSCADWAARWYFVASVGQCNRFWYGGCHGNANNFASEQECMSSC	Papilin	BPTI/Kunitz inhibitor	PAPLN
1knt_A	P12111	40.7	5	58	1	54	7.69E-05	221	PKFCLRPKNPGKCKQALKKFFFNAETSKCEEFIYGGCGGNKNNFDTKKECRLAC	TDICKLPKDEGTCRDFILKWYYDPNTKSCARFWYGGCGGNENKFGSQKECEKVC	Collagen alpha-3(VI) chain	BPTI/Kunitz inhibitor	COL6A3
AF-P02760-F1-model_v4	P02760	42.3	7	58	230	281	3.39E-05	246	FCLRPKNPGKCKQALKKFFFNAETSKCEEFIYGGCGGNKNNFDTKKECRLAC	SCQLGYSAGPCMGMTSRYFYNGTSMACETFQYGGCMGNGNNFVTEKECLQTC	Protein AMBP	BPTI/Kunitz inhibitor 2	AMBP
AF-O43278-F1-model_v4	O43278	35.8	7	59	390	442	9.79E-05	231	FCLRPKNPGKCKQALKKFFFNAETSKCEEFIYGGCGGNKNNFDTKKECRLACK	HCVDLPDTGLCKESIPRWYYNPFSEHCARFTYGGCYGNKNNFEEEQQCLESCR	Kunitz-type protease inhibitor 1	BPTI/Kunitz inhibitor 1	SPINT1

SI References

1. M. Steinegger, M. Mirdita, J. Söding, Protein-level assembly increases protein sequence recovery from metagenomic samples manyfold. *Nat Methods* **16**, 603–606 (2019).
2. M. Steinegger, J. Söding, Clustering huge protein sequence sets in linear time. *Nat Commun* **9**, 2542 (2018).
